# Deleterious *ABCA7* mutations and transcript rescue mechanisms in early onset Alzheimer’s disease

**DOI:** 10.1007/s00401-017-1714-x

**Published:** 2017-04-27

**Authors:** Arne De Roeck, Tobi Van den Bossche, Julie van der Zee, Jan Verheijen, Wouter De Coster, Jasper Van Dongen, Lubina Dillen, Yalda Baradaran-Heravi, Bavo Heeman, Raquel Sanchez-Valle, Albert Lladó, Benedetta Nacmias, Sandro Sorbi, Ellen Gelpi, Oriol Grau-Rivera, Estrella Gómez-Tortosa, Pau Pastor, Sara Ortega-Cubero, Maria A. Pastor, Caroline Graff, Håkan Thonberg, Luisa Benussi, Roberta Ghidoni, Giuliano Binetti, Alexandre de Mendonça, Madalena Martins, Barbara Borroni, Alessandro Padovani, Maria Rosário Almeida, Isabel Santana, Janine Diehl-Schmid, Panagiotis Alexopoulos, Jordi Clarimon, Alberto Lleó, Juan Fortea, Magda Tsolaki, Maria Koutroumani, Radoslav Matěj, Zdenek Rohan, Peter De Deyn, Sebastiaan Engelborghs, Patrick Cras, Christine Van Broeckhoven, Kristel Sleegers, On behalf of the European Early-Onset Dementia (EU EOD) consortium, Valentina Bessi, Silvia Bagnoli, Frederico Simões do Couto, Ana Verdelho, Laura Fratiglioni, Alessandro Padovani, Zdenek Rohan, Cristina Razquin, Elena Lorenzo, Elena Iglesias, Manuel Seijo-Martínez, Ramon Rene, Jordi Gascon, Jaume Campdelacreu, Rafael Blesa

**Affiliations:** 1 Neurodegenerative Brain Diseases Group, VIB Center for Molecular Neurology, VIB, Antwerp, Belgium; 2 Institute Born-Bunge, University of Antwerp, Antwerp, Belgium; 3 Department of Neurology, Antwerp University Hospital, Edegem, Belgium; 4 Department of Neurology and Memory Clinic, Hospital Network Antwerp (ZNA) Middelheim and Hoge Beuken, Antwerp, Belgium; 5 Alzheimer’s Disease and Other Cognitive Disorders Unit, Neurology Department, Hospital Clínic, Institut d’Investigacions Biomediques August Pi i Sunyer (IDIBAPS), Barcelona, Spain; 6 Department of Neuroscience, Psychology, Drug Research and Child Health (NEUROFARBA), University of Florence, Florence, Italy; 7 IRCCS Don Gnocchi, Florence, Italy; 8 Neurological Tissue Bank of the Biobanc, Hospital Clinic, Institut d’Investigacions Biomediques August Pi i Sunyer (IDIBAPS), Barcelona, Spain; 9 Department of Neurology, Fundación Jiménez Díaz, Madrid, Spain; 10 Memory Unit, Department of Neurology, University Hospital Mútua de Terrassa, University of Barcelona School of Medicine, Terrassa, Barcelona Spain; 11 Centro de Investigación Biomédica en Red de Enfermedades Neurodegenerativas (CIBERNED), Instituto de Salud Carlos III, Madrid, Spain; 12 Neuroimaging Laboratory, Division of Neurosciences, Center for Applied Medical Research (CIMA), University of Navarra, Pamplona, Spain; 13 Department of Neurology, Clínica Universidad de Navarra, University of Navarra School of Medicine, Pamplona, Spain; 14 Department of Neurobiology, Care Sciences and Society (NVS), Division of Neurogeriatrics, Center for Alzheimer Research, Karolinska Institutet, Huddinge, Stockholm, Sweden; 15 Genetics Unit, Department of Geriatric Medicine, Karolinska University Hospital, Stockholm, Sweden; 16 Molecular Markers Laboratory, Istituto di Ricovero e Cura a Carattere Scientifico (IRCCS), Istituto Centro San Giovanni di Dio-Fatebenefratelli, Brescia, Italy; 17 MAC Memory Center, Istituto di Ricovero e Cura a Carattere Scientifico (IRCCS), Istituto Centro San Giovanni di Dio-Fatebenefratelli, Brescia, Italy; 18 Faculty of Medicine, University of Lisbon, Lisbon, Portugal; 19 Neurology Unit, Department of Clinical and Experimental Sciences, Centre for Neurodegenerative Disorders, University of Brescia, Brescia, Italy; 20 Center for Neuroscience and Cell Biology, University of Coimbra, Coimbra, Portugal; 21 Department of Psychiatry and Psychotherapy, Technische Universität München, Munich, Germany; 22 Department of Neurology, IIB Sant Pau, Hospital de la Santa Creu i Sant Pau, Universitat Autònoma de Barcelona, Barcelona, Spain; 23 3rd Department of Neurology, Medical School, Aristotle University of Thessaloniki, Thessaloniki, Greece; 24 Laboratory of Biochemistry, Department of Chemistry, Aristotle University of Thessaloniki, Thessaloniki, Greece; 25 Department of Pathology, First Medical Faculty, Charles University, Prague, Czech Republic; 26 Department of Pathology and Molecular Medicine, Thomayer Hospital, Prague, Czech Republic; 27 Institute of Pathology, Third Medical Faculty, Charles University, Prague, Czech Republic

**Keywords:** Early Onset Alzheimer’s disease, ATP-Binding Cassette, Sub-Family A, Member 7 (ABCA7), Third-generation long-read sequencing, RNA sequencing, Loss-of-function, Modifier

## Abstract

Premature termination codon (PTC) mutations in the ATP-Binding Cassette, Sub-Family A, Member 7 gene (*ABCA7*) have recently been identified as intermediate-to-high penetrant risk factor for late-onset Alzheimer’s disease (LOAD). High variability, however, is observed in downstream *ABCA7* mRNA and protein expression, disease penetrance, and onset age, indicative of unknown modifying factors. Here, we investigated the prevalence and disease penetrance of *ABCA7* PTC mutations in a large early onset AD (EOAD)—control cohort, and examined the effect on transcript level with comprehensive third-generation long-read sequencing. We characterized the *ABCA7* coding sequence with next-generation sequencing in 928 EOAD patients and 980 matched control individuals. With MetaSKAT rare variant association analysis, we observed a fivefold enrichment (*p* = 0.0004) of PTC mutations in EOAD patients (3%) versus controls (0.6%). Ten novel PTC mutations were only observed in patients, and PTC mutation carriers in general had an increased familial AD load. In addition, we observed nominal risk reducing trends for three common coding variants. Seven PTC mutations were further analyzed using targeted long-read cDNA sequencing on an Oxford Nanopore MinION platform. PTC-containing transcripts for each investigated PTC mutation were observed at varying proportion (5–41% of the total read count), implying incomplete nonsense-mediated mRNA decay (NMD). Furthermore, we distinguished and phased several previously unknown alternative splicing events (up to 30% of transcripts). In conjunction with PTC mutations, several of these novel *ABCA7* isoforms have the potential to rescue deleterious PTC effects. In conclusion, *ABCA7* PTC mutations play a substantial role in EOAD, warranting genetic screening of *ABCA7* in genetically unexplained patients. Long-read cDNA sequencing revealed both varying degrees of NMD and transcript-modifying events, which may influence ABCA7 dosage, disease severity, and may create opportunities for therapeutic interventions in AD.

**Electronic supplementary material:**

The online version of this article (doi:10.1007/s00401-017-1714-x) contains supplementary material, which is available to authorized users.

## Introduction

Alzheimer’s disease (AD, MIM: 104300) is the most common form of dementia. More than 20 genomic loci have been identified to contribute to AD risk [17, 18, 26, 27, 37, 46]. Among those, the gene encoding ATP-Binding Cassette, Sub-Family A, Member 7 (*ABCA7*, MIM: 605414) is of particular interest, because both common variants and rare variants are reported to affect AD risk [11, 13, 16, 27, 41, 44, 47, 49]. ABCA7 plays a role in lipid metabolism [20, 32, 43, 48] and microglial phagocytosis [15, 21, 31], and was linked to altered amyloid β (Aβ) processing [23, 43, 45], the predominant hypothesis on AD pathogenesis.

Deleterious premature termination codon (PTC) mutations (nonsense, frameshift, and splice site mutations) in ABCA7 are observed at varying disease penetrance, with a 1.5–4× increased frequency in AD patients across populations [11, 16, 47, 49]. PTC mutation carriers appear more frequent among AD patients with a positive family history, though a wide range of disease onset age is observed [7, 11]. Two pedigrees have been reported in which a PTC mutation in *ABCA7* (p.Arg578fs and p.Glu709fs) co-segregates with disease [10, 11]. Although the mode-of-action of *ABCA7* PTC mutations in AD pathogenesis is unknown, a plausible mechanism is loss-of-function (LOF) due to nonsense-mediated mRNA decay (NMD). This is in line with mouse *Abca7* knockout experiments leading to increased Aβ brain levels [15, 23, 43]. Single-epitope quantification of human brain mRNA and protein levels of ABCA7 in PTC mutation carriers, however, is conflicting. High variability is observed between individuals in general and between PTC mutation carriers [1, 11]. Furthermore, mRNA and protein levels do not seem to correlate [1], necessitating analysis of *ABCA7* expression in a broader context. In addition to PTC mutations, rare predicted deleterious missense mutations and some common missense variants were, respectively, linked to risk increasing [16] and protective effects [11, 44], though requiring further confirmation.

The observation that *ABCA7* PTC mutations exert a relatively strong effect on individual risk and familial occurrence of AD warrants further exploration of their potential in individualized genetic diagnosis and risk prediction [12]. To address the current complexity on a clinical and molecular level, we examined the prevalence and characteristics of *ABCA7* coding mutations in a large European cohort of early onset AD patients (EOAD, onset age ≤65), a subgroup of AD patients that would strongly benefit from improved diagnosis, and genetic counseling. For a subset of PTC mutations, we performed targeted transcript analysis with third-generation (long-read) sequencing to gain further insight in the mode-of-action of these mutations and *ABCA7* dosage modifying events.

## Materials and methods

### Study population

The EOAD patients and control individuals were recruited within the European Early Onset Dementia (EU EOD) consortium. Details about recruitment, in- and exclusion criteria, and demographic and patient characteristics were previously described [51]. In summary, patients [*n* = 928, 60.2% (558/927) female, 51.2% (391/763) *APOE* ε4+ (MIM: 107741)] were diagnosed according to NINCDS-ADRDA [34] and/or NIA-AA [19, 35] diagnostic criteria, and had a mean onset age of 57.4 ± 5.6 years (30–65). In 47.7% (274/575) of patients, a positive familial history for dementia was reported. Patients were ascertained in neurological centers with an expertise in memory disorders, and originated from Spain (*n* = 403), Italy (*n* = 159), Sweden (*n* = 160), Germany (*n* = 83), Portugal (*n* = 66), Greece (*n* = 52), and the Czech Republic (*n* = 5) (Table S1). Seventeen EOAD patients carried a known pathogenic mutation in *PSEN1*, *PSEN2,* or *APP* (MIM: 104311, 600759, and 104760). Control individuals (*n* = 980, 64.9% (607/936) female) had a mean inclusion age of 63.9 ± 7.7 years (34-89), and were recruited in Spain (*n* = 223), Italy (*n* = 304), Sweden (*n* = 295), Portugal (*n* = 120), Greece (*n* = 35), and the Czech Republic (*n* = 3). The study was approved by the respective ethics committees, and all participants and/or their legal guardian provided written informed consent before inclusion.

### *ABCA7 s*equencing

Genomic DNA (gDNA) was amplified with Illustra GenomiPhi v2 (Thermo Fisher, Waltham, MA, USA). Enrichment of 43 of the 47 canonical *ABCA7* (RefSeq NM_019112.3) exons and splice sites was based on a custom multiplex PCR assay (primer sequences available upon request) generated with mpcr software (Multiplicom, Niel, Belgium). Regions of interest were amplified using flanking primers with universal adapter sequences [5′-TCGTCGGCAGCGTCAGATGTGTATAAGAGACAG-(sequence-specific forward primer) and 5′-GTCTCGTGGGCTCGGAGATGTGTATAAGAGACAG-(sequence-specific reverse primer)]. Amplicons were PCR barcoded with Nextera XT sequences (Illumina, USA) targeting previously incorporated adapters. Samples were 2 × 300 bp paired-end sequenced with MiSeq Reagent Kit v3 (Illumina, San Diego, CA, USA). Adapter clipping of demultiplexed FASTQ output was performed with fastq-mcf [3], aligned with Burrows-Wheeler Aligner MEMv0.7.5a [30] and variants were called with GATKv2.4 UnifiedGenotyper and GATKv3.5 HaplotypeCaller. Variants were annotated with GenomeComb [42] and SnpEff [9].

**Fig. 1:**
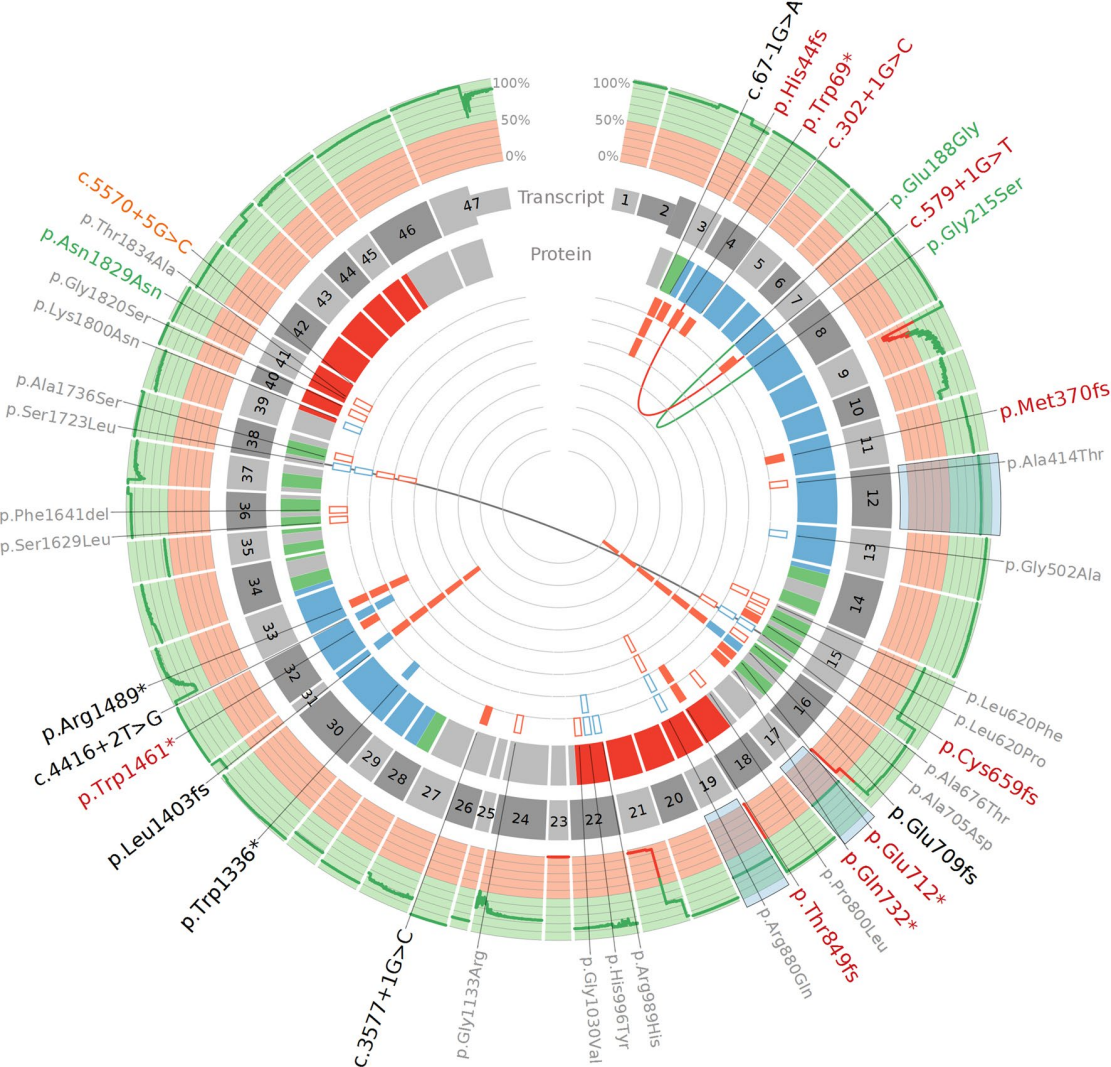
*ABCA7* mutation screening in EOAD. From the outside to the inside: HGVS nomenclature is denoted for known (*black*) and novel (*red*) PTC mutations, suggestively associated protective common variants (*green*), intron retaining variant c.5570+5G>C (*orange*), and predicted deleterious missense variants (*gray*). The second rim corresponds to the percentage of included individuals covered at more than 20× on the corresponding exonic position (0–50% in *red* and 50–100% in *green*). Exons highlighted in blue were screened with Sanger sequencing. The third layer consists of the UTR (narrow) and CDS (broad) architecture of *ABCA7* (NM_019112.3). Next, the corresponding predicted protein domains (UniProtKB entry: Q8IZY2) are shown (transmembrane domains in *green*, extracellular parts in *blue* and *red* corresponds to ABC domains). In the *center*, the number of carriers per PTC (*filled bars*) and deleterious missense (*open bars*) variant are shown. Patients are represented in *red*, while control individuals are shown in *blue*. Linked variants (*red*, *green*, and *gray line*) segregated on the same haplotype

For four exons (12, 17, 19, and 23), no compatible primers were found. Exons 12, 17, and 19 were analyzed with Sanger sequencing: exons were PCR amplified, subsequently dideoxy-terminated with BigDye termination cycle sequencing kit v3.1 (Thermo Fisher), and sequenced with ABI3730 DNA Analyzer (Thermo Fisher). Sanger sequences were analyzed using Seqman (DNAstar, Madison, WI, USA) and NovoSNP software [52]. Exon 23 (73 bp + 4 bp splice sites) was not duly screened. Of note, this exon contains no known PTC mutations [Exome Aggregation Consortium (ExAC) (accessed April 2017)]. In addition, gaps are present (less than 50% of individuals sequenced at 20× coverage) in exon 9 (29 bp), 16 (23 bp), 18 (4 bp), and 21 (87 bp), as depicted in Fig. 1, mainly due to repetitive regions flanking *ABCA7* exons, limiting efficient PCR primer design.

The sequencing read-depth statistics for all coding and splice sites regions are represented in Fig. 1 based on Rsamtools [36] and Circos visualization [25]. All PTC variants and c.5570+5G>C were validated on gDNA with Sanger sequencing as described above. In addition, we validated rare [minor allele frequency (MAF) <0.01%] missense variants with at least 20× read depth, a less than threefold difference between reference and alternative allele counts for heterozygous variants, and a Phred-scaled CADD score [24] above 20, corresponding to the 1% most deleterious variants in the human genome. This cutoff was chosen given the relatively high mutational tolerance of *ABCA7* and incomplete disease penetrance for very deleterious PTC mutations.

### Association analysis

Rare variant (MAF < 0.01) association meta-analysis by ethnicity was performed separately for PTC and predicted deleterious missense mutations using heterogeneous genetic effect SKAT-O statistics within the MetaSKAT framework [29]. *APOE* dosage (number of ε4 alleles) was tested as a covariate. Summary odds ratios (OR_MH_) were calculated with a Cochran–Mantel–Haenszel test in PLINK [40], stratified by country of origin, on individuals without missing genotyping information.

For common variants (MAF ≥ 0.01), a fixed-effects (Cochran–Mantel–Haenszel) meta-analysis was used to calculate single variant allelic association. We evaluated all SNPs located within the CDS and 15 bp exon flanking regions passing Hardy–Weinberg equilibrium quality control (*p* > 0.001). Odds ratios are reported for the minor allele with 95% confidence intervals. Pairwise linkage disequilibrium (LD) was calculated in PLINK [40]. To account for LD between variants, multiple testing thresholds were based on spectral decomposition [38], leading to a study-wide multiple testing *p* value cutoff of 0.0033. Carriers of a known pathogenic mutation in *APP*, *PSEN1,* or *PSEN2* were excluded from association analyses.

### Transcript analysis of PTC mutation carriers

Fresh frozen brain was available for patient EOD-P1 (c.67-1G > A mutation). RNA was extracted from anterior cingulate cortex tissue with Ambion RiboPure™ kit (Life Technologies, Carlsbad, CA, USA). Reagent volumes were adapted to support 25 mg of input brain material. In addition, RNA from blood was extracted with Tempus™ blood RNA tube (Thermo Fisher) for carriers EOD-P7 (p.Met370fs) and EOD-P19 (c.3577+1G>C). For a subset of *ABCA7* PTC mutations, EBV-transformed lymphocytes (p.Glu709fs, p.Trp1336*, and c.5570+5G>C) or fresh frozen brain tissue (p.Leu1403fs) from AD patients was available through the BELNEU consortium [11]. RNA was extracted with Ambion RiboPure™ kit (Life Technologies). All RNA was treated with Ambion TURBODNase kit (Life Technologies) to degrade potential gDNA contamination. First-strand cDNA was synthesized with SuperScript^®^III First-Strand Synthesis System (Life Technologies).

We performed cDNA sequencing on a MinION platform (Oxford Nanopore Technologies, Oxford, UK). Exonic primers were designed with Primer3Plus to generate amplicons containing exons of interest, flanked by at least one splicing event on each side (Table S2). All PCR amplifications were performed with 35 cycles to have sufficient amplification of the lowly expressed ABCA7 in all tissues. Titanium Taq (Clontech Laboratories, Mountain View, CA, USA) or Platinum Taq (Thermo Fisher) enzymes were used with or without supplementation of betain, depending on the GC content of the amplicon. In case of overlapping PCR amplicons, a 5′ barcoding adapter sequence was added to the primers and amplicons were barcoded with the PCR 96 barcoding genomic DNA (R9) kit (Oxford Nanopore Technologies) using 15 amplification cycles on a 1/125 diluted template. DNA was purified with Agencourt AMPure XP SPRI beads (Beckman Coulter, Brea, CA, USA) and concentrations were measured with Qubit (Thermo Fisher). Amplicons were then pooled equimolar to a total quantity of 0.2 pmol. The resulting PCR library was then further prepared according to the manufacturer’s protocol. Briefly, DNA was treated with NEBNext Ultra II End Repair/dA-Tailing Module (New England BioLabs, Ipswich, MA, USA). The product was then purified with AMPure XP beads, after which the ONT sequencing adapter and hairpin were ligated with the NEB Blunt/TA Ligase Master Mix (New England BioLabs). Next, biotin containing hairpin tethers were added and washed MyOne Streptavidin C1 Dynabeads (Thermo Fisher) were used for pull-down. Finally, the sequencing library was eluted from the beads and supplemented with Running Buffer with Fuel Mix. SQK-MAP006/NSK007 chemistry and FLO-MAP103/MIN104 flow cells were used for sequencing. Base calling was done with the 2D plus barcoding protocol (Metrichor, Oxford, UK) after which FASTQ sequences were extracted with poretools v0.6.0 [33] according to the “best” protocol. Sequencing reads were aligned to the human genome (hg19) with GMAP v2016-06-30 [53] to account for splicing events. Wild-type (WT), nonsense, and rescue alleles were quantified with Rsamtools v1.26.0 for aligned sequences [36], and with GMAP splicesites output for splicing events. The PTC expression fraction was calculated as (expression_Nonsense_/expression_Nonsense+WT_); 0% corresponds to complete degradation of PTC bearing transcripts and 50% to equal observance of PTC and WT transcripts. Results were validated with Sanger sequencing of cDNA amplicons as described above and compared with RNA sequencing (RNAseq) data from a Belgian unrelated AD-control transcriptome study [51]. Briefly, total RNA was isolated from Epstein–Barr virus immortalized lymphoblasts derived from whole blood lymphocytes. RNA isolation of lymphoblast cells was performed with the RNeasy mini kit (Qiagen Inc., Valencia, CA, USA) according to the manufacturer’s protocol. Sequence libraries were constructed using the Truseq stranded mRNA Library Prep Kit v2 (Illumina) using 1 mg total RNA for each sample. Sequencing of prepared libraries was performed using an Illumina HiSeq 2000 sequencer generating an average of 72 × 106 ± 6 × 106 paired-end sequence reads/sample. Subsequent data processing consisted out of removal of read adapters and trimming of read ends with Trimmomatic [6]. Reads were then aligned to hg19 using the Bowtie short read aligner integrated in Tophat2 [22].

## Results

### PTC mutations

Sequencing of the *ABCA7* CDS in 928 EOAD patients and 980 control individuals revealed 17 different PTC mutations (six frameshift indels, six nonsense mutations, and five PTC-introducing splice site mutations) (Table 1), which were more frequent in EOAD patients (3.02%; *n* = 28) than controls (0.61%; *n* = 6) [*p* value = 0.0004, OR_MH_ = 5.01 (95% CI = 1.59–15.72)]. This association remained significant after correction for *APOE* (p = 0.001). Most PTC mutations (*n* = 10) were not reported before and—together with c.67-1G > A, c.3577 + 1G > C, and p.Arg1489*—were absent from control individuals (Fig. 1; Table S3). Patient EOD-P5 carried two PTC mutations (p.Trp69*and c.579+1G>T) segregating on the same haplotype (Figure S1). Two previously reported variants (p.Trp1336*, c.4416+2T>G) were only observed in control individuals. The two most frequent PTC mutations (p.Glu709fs and p.Leu1403fs) were observed more in patients than control individuals (Fig. 1; Table 1). All carriers of p.Glu709fs and p.Leu1403fs shared the same respective haplotype (Table S4). Carriers of less frequent multiple occurring variants (c.67-1G>A, p.Thr849fs, c.4416+2T>G, and p.Arg1489) were geographically confined (respectively, originating from Spain, Portugal, Italy, and the Iberian Peninsula), though no familial relatedness was known (Table S3).

**Table 1:** *ABCA7* PTC mutations

Genomic position	HGVS (coding)	HGVS (protein)	dbSNP	Patient carriers (MAF)	Control carriers (MAF)	Previous reports	PTC expression fraction	Reading frame restoration (expression fraction)
PTC mutations								
chr19:1041508	c.67−1G>A	–	rs199517248	3 (0.16%)	0 (0%)	1, 3	5%	–
chr19:1041565	c.124_130dupGTTCGCC	p.His44fs	–	1 (0.05%)	0 (0%)	Novel	–	–
chr19:1041875	c.206G>A	p.Trp69*	–	1 (0.05%)	0 (0%)	Novel	–	–
chr19:1041972	c.302+1G>C	–	–	1 (0.05%)	0 (0%)	Novel	–	–
chr19:1042826	c.579+1G>T	–	–	1 (0.05%)	0 (0%)	Novel	–	–
chr19:1044636	c.1109dupT	p.Met370fs	–	1 (0.06%)	0 (0%)	Novel	21%	Exon skipping (2%)
chr19:1047275	c.1968_1977delTGCGGCCTGC	p.Cys659fs	–	1 (0.07%)	0 (0%)	Novel	–	–
chr19:1047507	c.2126_2132delAGCAGGG	p.Glu709fs	rs547447016	6 (0.36%)	2 (0.12%)	1, 2, 3, 4	41%	Alternative splicing (10%)
chr19:1047518	c.2134G>T	p.Glu712*	–	1 (0.06%)	0 (0%)	Novel	–	–
chr19:1047578	c.2194C>T	p.Gln732*	–	1 (0.06%)	0 (0%)	Novel	–	–
chr19:1049426	c.2544delC	p.Thr849fs	–	2 (0.11%)	0 (0%)	Novel	–	–
chr19:1054110	c.3577+1G>C	–	rs373195428	1 (0.05%)	0 (0%)	3	36%	–
chr19:1055153	c.4008G>A	p.Trp1336*	–	0 (0%)	1 (0.05%)	3	27%	Exon skipping (8–30%)
chr19:1055907	c.4208delT	p.Leu1403fs	rs538591288	5 (0.27%)	1 (0.06%)	1, 2, 3, 4	38%	Exon skipping (3–4%)
chr19:1056208	c.4382G>A	p.Trp1461*	–	1 (0.05%)	0 (0%)	Novel	–	–
chr19:1056244	c.4416+2T>G	–	rs113809142	0 (0%)	2 (0.10%)	2, 3, 4	–	–
chr19:1056377	c.4465C>T	p.Arg1489*	–	3 (0.17%)	0 (0%)	3	–	–
Splice affecting mutation								
chr19:1061892	c.5570 + 5G>C	–	rs200538373	6 (0.38%)	7 (0.38%)	1, 2, 3	30%	–

An overview of all premature termination codon (PTC) inducing mutations observed in this study. Genomic coordinates are based on hg19. HGVS = mutation nomenclature according to the Human Genome Variation Society. dbSNP notations refer to Reference SNP IDs (rs) from dbSNP build 142. *MAF* minor allele frequency. The previous reports of the mutation in discovery populations of Cuyvers et al. (1), Steinberg et al. (2), Le Guennec et al. (3), or Nuytemans et al. (4) are denoted. Abundance of the PTC transcript is shown based on MinION cDNA sequencing (Figures S3–S5, S8, and S10). PTC expression fraction corresponds to the abundancy of sequencing reads containing the PTC mutation, in which case 0% is associated with complete degradation of PTC transcripts, and 50% with equal expression of PTC and WT alleles. Reading frame restorations are denoted when identified and correspond either to in-frame skipping of the exon harboring the mutation of interest, or alternative splicing which restores the reading frame without introduction of a PTC. The same cDNA PCR amplicon was used to measure exon skipping events for p.Trp1336* and p.Leu1403fs; as a result, both exons could be quantified for two different individuals (range)

Patients (*n* = 28, 75.0% female, 54.2% (13/24) *APOE* ε4 +) carrying PTC mutations had a mean onset age of 56.7 ± 5.5 years (42-65, upper limit determined by inclusion criteria for EOAD), and a mean disease duration of 8.6 ± 1.8 years (Table S3). In comparison, carriers of established pathogenic mutations in *PSEN1* (*n* = 12), *PSEN2* (*n* = 3), or *APP* (*n* = 2) had a lower mean onset age of, respectively, 46.0 ± 9.0, 53.0 ± 5.1, or 49.5 ± 1.5 years (35-63, p = 0.0002) (Figure S2). Patient EOD-P6.1 (*ABCA7* c.302 + 1G>C) also carried a pathogenic *PSEN1* mutation (p.His163Arg) and had the earliest onset age (42 years) of all *ABCA7* PTC carriers. Two affected relatives of EOD-P6.1 also carried *PSEN1* p.His163Arg, but not *ABCA7* c.302+1G>C, and had a slightly older onset age of 46 years.

**Fig. 2:**
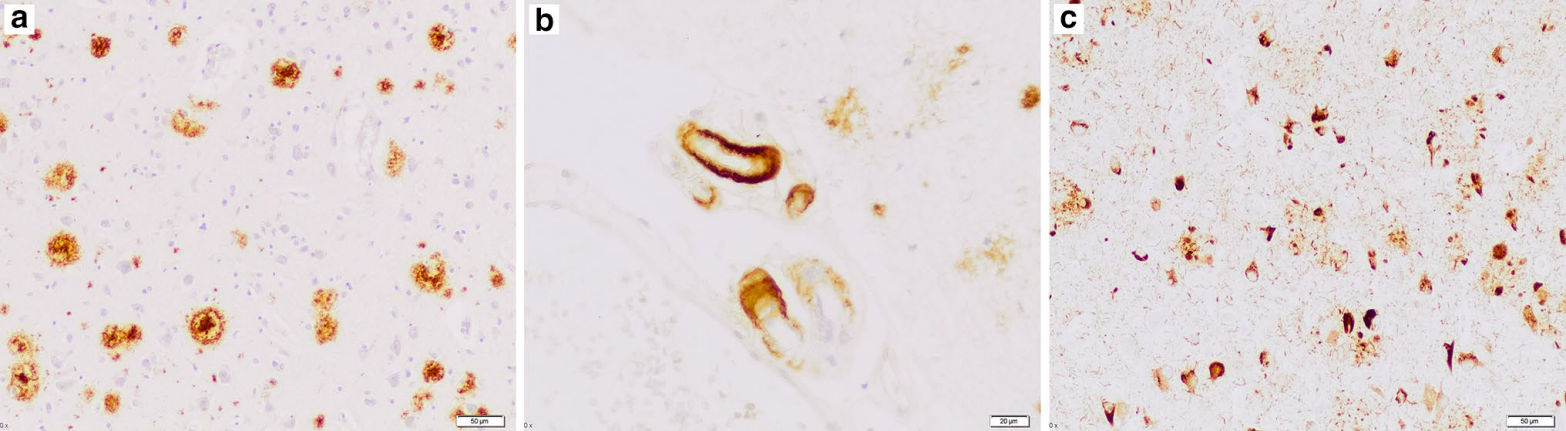
Neuropathological findings in patient EOD-P1. Abundant beta-A4 amyloid pathology in the form of diffuse and cored amyloid plaques (**a**) and amyloid angiopathy involving leptomeningeal vessels (**b**). Prominent phospho-tau pathology in the form of neurofibrillary tangles, dystrophic neurites, and neuropil threads (**c**). *Scale bars*: **a, c** 50 μm, **b** 20 μm. An extended neuropathological description is available in Neuropathological description S1

A positive familial history for dementia was reported in 61.5% (16/26) of patient carriers. Of note, for patient EOD-P21.1 carrying p.Leu1403fs, DNA was available of two affected relatives with onset ages of 68 and 70 years, who both also carried the mutation. In addition, EOD-P7 (p.Met370fs) and EOD-P20 (p.Leu1403fs) had a negative familial history for dementia, but both had a first-degree relative with Parkinson’s disease. Information on clinical presentation was available for 23 patients, of whom 82.6% (19/23) had a predominant amnestic presentation. In two patients, the onset of memory dysfunction was accompanied by language dysfunction. EOD-P6.1 (carrying *PSEN1* p.His163Arg) had prominent behavioral symptoms (aggressiveness). Only one patient presented with a clear nonamnestic phenotype (logopenic progressive aphasia). Neuropathology was available for EOD-P1, confirming the clinical AD diagnosis (Fig. 2, Neuropathological description S1).

### Transcript analysis of PTC mutations

**Fig. 3:**
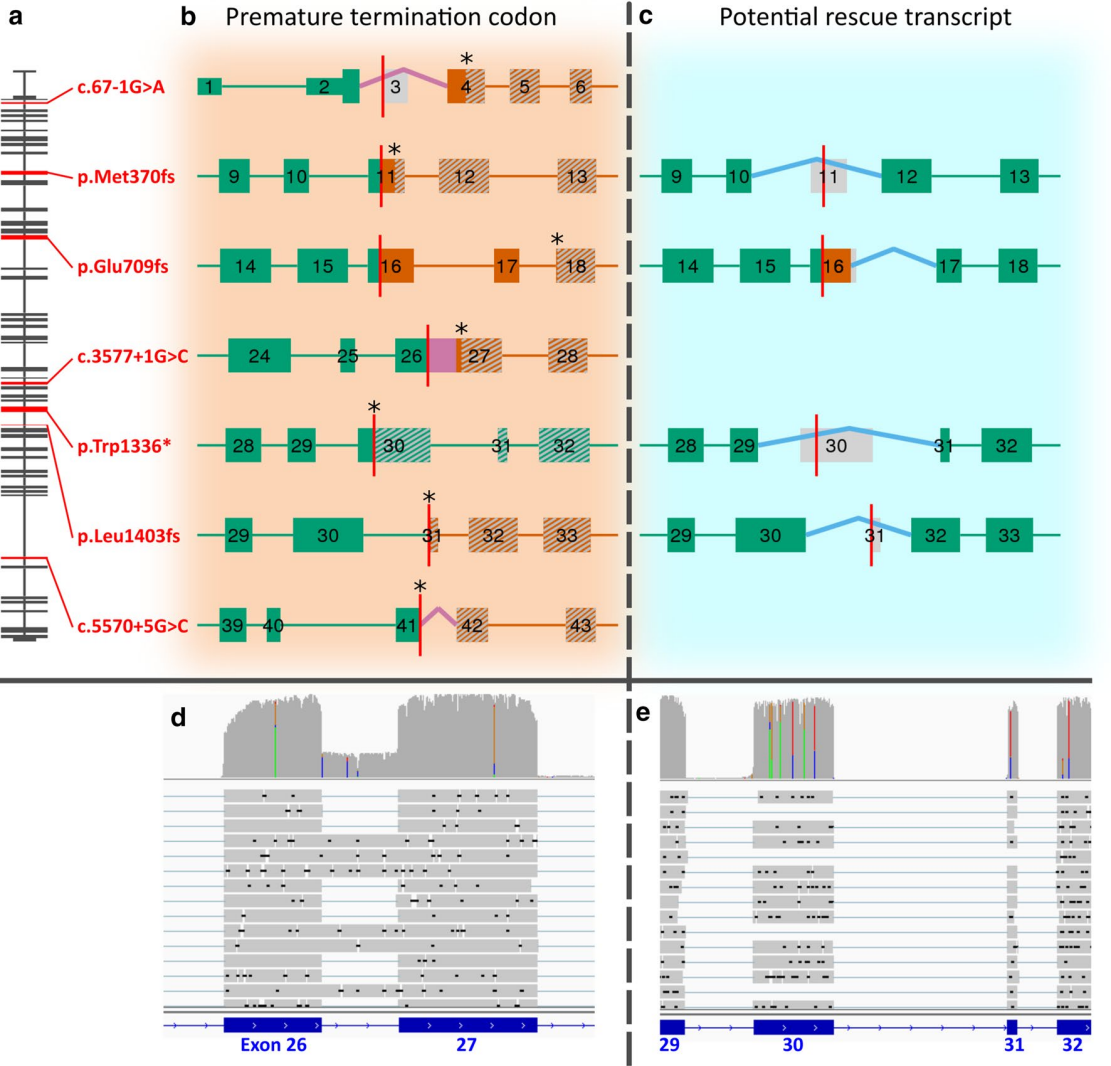
Schematic representation of PTC mutations and their effect on transcripts as well as corresponding potential rescue mechanisms. **a** The canonical *ABCA7* transcript is shown from exon 1 (*top*) to exon 47 (*bottom*). Exons in red harbor a PTC inducing mutation (HGVS notation in *red*) which was analyzed on transcript level. Observed transcripts generated through MinION cDNA sequencing of patient cDNA are shown in two *panels*: NMD escaping transcripts harboring PTC mutations (**b**) and potential rescued transcript through alternative splicing (**c**). PTC inducing mutations (*vertical red line*), transcribed regions (broad, *numbered segments*) and connecting nontranscribed introns (*horizontal lines*), are shown. The transcript reading frame is either in-frame (*green*) or out-frame (*orange*). The first induced PTC mutation is denoted with an *asterisk*. Downstream transcript which is not translated and results in truncated ABCA7 protein is shaded in *gray*. Exons completely in *gray* are skipped and alternative splicing is shown as caret-like connectors, or as transcribed fragments in the case of intron retention. Alternative splicing events can either be deleterious (*pink*), or may potentially rescue transcripts (*blue*). Raw sequencing data supporting alternative splicing are shown for two cases in (**d**) and (**e**): Overall, read depth per position is represented on *top* as a *bar chart* (*gray* for reference nucleotides, different *colors* for SNPs). Separate long sequencing reads are shown below (*gray bars* for aligned sequences, *blue lines* for connecting splicing events, and *black lines* for deletions), and the exonic layout of *ABCA7* is depicted at the *bottom* (*blue bars*). **d** MinION cDNA sequencing of a patient carrying c.3577 + 1G > C confirms complete out of frame retention of intron 26. **e** MinION cDNA sequencing confirms that both exon 30 and 31 can be skipped in-frame and can, therefore, alter the effect of PTC mutations positioned in these exons (i.e., p.Trp1336* and p.Leu1403fs, respectively)

We examined *ABCA7* expression with MinION sequencing for three frameshift mutations (p.Met370fs, p.Glu709fs, and p.Leu1403fs), one nonsense (p.Trp1336*), one splice donor (c.3577 + 1G > C), and one splice acceptor mutation (c.67-1G>A). We observed varying degrees of PTC bearing transcripts in all cDNA libraries (Fig. 3; Table 1), indicative of incomplete NMD. The most N-terminal mutation (c.67−1G>A) had the highest NMD efficiency with only 5% of sequencing reads showing out-of-frame exon 3 skipping. All other mutations presented higher PTC abundancy (21–41%), approaching expression equal to the WT allele (50%). On top of apparent NMD escape, we observed alternative splicing events, absent from public databases (e.g., Ensembl, GENCODE, and UCSC genes), in mutated regions of interest. Some have the ability to restore reading frameshifts caused by PTC mutations (Fig. 3; Table 1). For three mutations (p.Met370fs, p.Trp1336*, and p.Leu1403fs), in-frame skipping of the respective PTC bearing exon [exon 11 (168 bp), exon 30 (255 bp), and exon 31 (33 bp)] was observed in patient carriers (Figures S3, S4, and S5). Overall, potential PTC rescue transcripts had a modest abundance (2–8%); however, in brain cDNA of one individual, we observed skipping of exon 30 in 30% of all sequencing reads, in this case unrelated to a PTC (Figure S5). These in-frame exon skipping transcripts were validated with RNAseq, confirming their presence in individuals not carrying an *ABCA7* mutation (Figure S6 and S7). Furthermore, for p.Glu709fs (exon 16), we observed usage of a cryptic splice donor site (10%) in the same exon (Figure S8), which can negate the frameshift effect of p.Glu709fs. Validation of this splicing isoform with RNAseq is presented in Figure S9, along with confirmation of its presence in a physiological context. Western blotting was performed on brain of c.67−1G>A and p.Leu1403fs (Method S1), confirming a reduction of expression by PTC mutations (Figure S11), as well as an overall variability in expression. One splice mutation (c.5570+5G>C)—3 bp downstream of the canonical splice donor site—was previously reported to cause out-of-frame intron retention [47]. Here, this variant showed no association: we observed the minor C-allele in six EOAD patients (MAF = 0.38%), of which one was homozygous, and seven control individuals (MAF = 0.38%) [OR_MH_ = 1.28 (0.44–3.74), *p* value = 0.86; Table S5]. RNAseq and MinION sequencing confirmed out-of-frame partial intron 41 retaining capability of c.5570+5G>C, but additional splicing events in the locus were observed as well (i.e., exon 41 skipping, and varying intron 41 retention lengths) (Figure S10).

### Other coding variants

We identified 19 predicted deleterious missense mutations (CADD score >20). There was no enrichment of predicted deleterious missense variants in patients (1.7%; *n* = 16) compared to controls (0.92%; *n* = 9; SKAT-O *p* value = 0.67) (Fig. 1; Table S6). Of note, p.Ala676Thr and p.Ser1723Leu segregated together in two controls and one patient; for which the patient, in addition, carried a third deleterious in-frame deletion (c.4922_4924delTCT). Clinical characteristics of missense mutation carriers did not differ substantially from PTC mutation carriers [mean onset age 57.0 ± 5.5 years (range = 46–55); positive familial history in 60.0% (6/10)].

Twenty-two common coding variants (MAF > 1%), were assessed for association with EOAD. No variants passed study-wide multiple testing correction (*p* = 0.0033), but nominal significance was observed for three SNPs (Table S7). The strongest effect [OR = 0.60 (95% CI = 0.42–0.87), *p* = 0.006] was observed for p.Gly215Ser (rs72973581, MAF = 4.1%), a missense SNP previously suggested to be protective [44]. For silent variant p.Asn1829Asn (rs78320196, MAF = 4.7%), we observed an OR of 0.65 (95% CI = 0.47–0.90; *p* value = 0.009). The potential protective effect of p.Asn1829Asn seems independent of p.Gly215Ser, given low pairwise LD (*R* ^2^ = 0.001 and *D*′ = 0.596). The third variant (p.Glu188Gly, rs3764645, and MAF = 43.4%) also showed a suggestive protective effect (OR = 0.86 95% CI = 0.75–0.99), *p* value = 0.030), which was previously reported in a Belgian cohort of AD [11]. We further observed that p.Glu188Gly and p.Gly215Ser were in strong LD (*D*′ = 0.961).

## Discussion

Predicted LOF mutations in *ABCA7* were recently put forward as intermediate-to-high penetrant risk factors for AD. To evaluate their contribution to EOAD, we sequenced the *ABCA7* coding DNA of 928 European EOAD patients and 980 ethnically matched healthy control individuals. We identified ten novel patient-specific PTC mutations (frameshift, nonsense, and canonical splice mutations), and confirmed seven previously reported mutations. *ABCA7* PTC mutations were five times more frequent among EOAD patients than controls, confirming an important contribution of these mutations to AD. Transcript analysis of seven PTC mutations revealed varying degrees of loss-of-transcript, suggesting that the mechanism through which these mutations affect AD risk needs further investigation. We observed no associations for predicted deleterious missense mutations, but detected a protective trend for three common variants.

*ABCA7* PTC mutations were detected in approximately 3% of the EOAD patients, which is comparable to the previous reports [11, 16]. In comparison, predicted pathogenic PTC mutations in *SORL1* (MIM: 602005)—another prominent AD risk gene—were observed in 0.6% of EOAD patients in the EU EOD consortium [51]. Furthermore, only an estimated 5–10% of EOAD can be explained by autosomal dominant mutations in *APP* (<1%), *PSEN1* (6%), and *PSEN2* (1%) [8]. Hence, due to relative high penetrance and occurrence, genetic screening for *ABCA7* PTC mutations is warranted in genetically unexplained EOAD patients. In line with the previous reports, we observed a high familial load in patients carrying an *ABCA7* PTC mutation—though lower than in established autosomal dominant mutation carriers. In one Italian AD family (EOD-P21), multiple affected relatives carried the *ABCA7* p.Leu1403fs mutation. Further elucidation of age-related penetrance and segregation patterns of individual *ABCA7* mutations—and possible modifiers thereof—will be imperative for implementation of *ABCA7* mutation screening in clinical practice and genetic counseling.

Based on the previous reports and the arbitrary positioning of AD associated PTC mutations across the gene (Fig. 1), haploinsufficiency—a reduction of dosage sensitive functional ABCA7—is the most plausible pathogenic mechanism [11, 16, 47]. Additional expression differences—either dosage recovery or further ABCA7 depletion—are, therefore, potential modifiers. We examined the effect of frameshifts, nonsense, splice donor, and splice acceptor variants on *ABCA7* transcripts in patient biomaterials using “third-generation” long-read MinION cDNA sequencing. Even though this sequencing technology is still under development and currently produces a relatively high random base calling error rate, we show that the accuracy is sufficient to align reads and to identify splicing events. Furthermore, given the high read depth attained in this targeted experiment (at least 1400×), reliable consensus sequences and variant calls could be formed.

Interestingly, we observed PTC transcripts for all mutations under study, indicating NMD escape. The proportion of sequencing reads carrying a PTC varied across mutations, up to 41% which is close to no NMD (50%). As a consequence of NMD escape, ABCA7 dosage may be modified, either via natural PTC read-through resulting in full length protein [5], or on the other hand through formation of truncated proteins which could exert dominant negative or wild-type functions. NMD escape also opens a window for pharmacological intervention. Several compounds are known to cause ribosomal read-through of PTCs, which could result in a functional protein and alleviate haploinsufficiency. Especially carriers of nonsense mutations may benefit from such a treatment. Read-through compounds (e.g., PTC124) are currently tested in clinical trials for LOF diseases such as Duchenne muscular dystrophy (DMD, MIM: 310200) and cystic fibrosis (MIM: 219700) [5].

In contrast to standard RNAseq, MinION sequencing of long DNA fragments at high read depth provided insights in phasing of mutations and splicing events despite low *ABCA7* expression [39]. As a result, we observed alternative splicing events unknown to public repositories. We identified cryptic splice site usage, often leading to a shift in reading frame, as well as exon skipping, both in- and out-of-frame. On one hand, these events can lower the ABCA7 cell reserve resulting in stronger dosage depletion when a PTC mutation is introduced. On the other hand, several splicing events have the potential to recover the effect of PTC causing mutations (e.g., reading frame rescue through usage of a cryptic splice site), or alter the amount of transcript carrying a PTC mutation (e.g., in-frame skipping of an exon harboring a mutation). Interestingly, for all PTC mutations observed in controls with the exception of c.4416+2T>G for which no biomaterials were available, a potential rescue mechanism was present (Table 1). For some mutations, the rescue event appears relatively frequent, which may contribute to incomplete penetrance (e.g., p.Trp1336* has been reported in both patients and controls; we observed exon 30 skipping in up to 30% of reads). Stabilization of alternative isoforms (e.g., through oligonucleotides targeting pre-mRNA) is a potential pharmacological target, which is already being evaluated for diseases as DMD and spinal muscular atrophy (MIM: 253300) [14].

Further research into the functions, essential protein domains, expression, and different isoforms of ABCA7 will have to substantiate to which extent dosage can modify the AD phenotype, and whether it can be remediated. It is likely that numerous factors contribute significantly to variation in ABCA7 expression (as evidenced by protein levels in hippocampus, Figure S11), including brain degeneration, inflammation, specific brain regions/cellular composition, disease duration, genetic etiology, and environmental factors. The long-read cDNA sequencing approach used here shows that differences in ABCA7 transcript and protein expression data may also partly be explained by a myriad of NMD escaping alternatively spliced transcripts and (truncated) proteins. Taken together, this may explain discrepancies within and between the previous studies on *ABCA7* expression [1, 2, 50]. In this study, *ABCA7* transcripts of PTC mutations were examined in different patient tissues (brain, blood, and lymphoblast), which may present varying NMD efficiencies [54] and alternative splicing [4]. Ideally, quantitative comparisons of *ABCA7* dosage between carriers are performed on a larger series of mutation carriers in a single tissue to more precisely determine the contribution of NMD efficiency and transcript rescue to variation in ABCA7 gene expression. In this study, we aimed at adequate coverage of lowly abundant *ABCA7* transcripts by sequencing mutation-specific amplicons, which, in addition, also prevents the formation of chimeric PCR molecules that might lead to phasing errors [28]. When current limitations of long-range PCR are overcome, it will be of interest to expand the methods used here to obtain a detailed map of transcript events across full length *ABCA7* mRNA.

In addition to canonical PTC mutations, c.5570 + 5G > C is known to cause out-of-frame intron retention [47], which we confirm (Figure S10). While others have observed association with this variant [16, 47], here, no enrichment was present (OR_MH_ = 1.28 95% CI = 0.44–3.74), *p* value = 0.86). Possibly, c.5570+G>C has a different protein reducing effect than canonical PTC mutations, since the degree of cryptic splice donor site versus canonical usage is unknown, and due to the relatively distal location of c.5570+5G>C in the protein. Furthermore, several interfering isoforms were present, suggesting lower penetrance of this particular variant. A previous report also suggested the pathogenicity of predicted damaging *ABCA7* missense variants [16]. In this study, with a larger study population, however, we observed no obvious enrichment (*p* = 0.66). Furthermore, we observed two deleterious missense variants (p.Ala676Thr and p.Ser1723Leu) segregating on the same haplotype, which occurred in both patients and controls. We cannot exclude that our cohort lacked power to observe a likely smaller effect of missense mutations on disease risk, but at this point, it is premature to draw inferences based on deleteriousness predictions alone. If future studies reveal a risk increasing effect of (a subset of) *ABCA7* missense variants, it may be worthwhile to elucidate the effect of these mutations on mRNA splicing and vice versa.

Finally, three common coding variants (p.Gly215Ser, p.Glu188Gly, and p.Asn1829Asn) showed a trend towards decreased risk of EOAD, albeit not withstanding multiple testing. Of note, p.Gly215Ser was previously put forward as protective variant in *ABCA7* [44], and for p.Glu188Gly and p.Asn1829Asn, a nominal protective association was also observed before [11, 44]. We show that p.Gly215Ser and p.Glu188Gly shared the same haplotype background (*D*′ = 0.961). In this study, we extend potential protecting effects of p.Gly215Ser and p.Asn1829Asn towards EOAD, supporting the role of *ABCA7* to mediate risk of (early onset) AD in both directions. Further research, however, is required to understand the downstream protective mechanisms.

In summary, with this targeted resequencing of *ABCA7* in a large European cohort of EOAD, we substantiate the evidence that *ABCA7* PTC mutations contribute significantly to AD risk. We observed a fivefold enrichment of *ABCA7* PTC mutations in EOAD patients, and provided further evidence that these mutations may segregate with disease in pedigrees. This suggests that at least some *ABCA7* mutations may have a high penetrance, providing new inroads for genetic subtyping and risk prediction. The observation of these ‘familial’ *ABCA7* mutations in cognitively healthy individuals, however, warrants cautious interpretation and further exploration of pathogenicity and modifying factors. An initial characterization of different PTC mutations at transcript level reveals substantial variability in NMD and alternative splicing, implying varying abundancy of ABCA7 in PTC mutation carriers. Further investigation is required into the degree of dosage reduction caused by a single mutation, the function and structure of ABCA7, and the presence of potential dominant negative effects, to contribute to a better estimation of phenotypical consequences and ways to remediate this.

## Electronic supplementary material

Below is the link to the electronic supplementary material.

Supplementary material 1 (PDF 936 kb)
