# Supplementary material for: Deleterious ABCA7 mutations and transcript rescue mechanisms in early onset Alzheimer’s disease
Source: Acta Neuropathol. 2017 Apr 27;134(3):475–87. doi: 10.1007/s00401-017-1714-x (PMC5563332; doi:10.1007/s00401-017-1714-x)
Supplement: Supplementary file 1 — Supplementary material 1 (PDF 936 kb) [file 401_2017_1714_MOESM1_ESM.pdf]

Deleterious *ABCA7* mutations and transcript rescue mechanisms in early-onset Alzheimer's disease

Arne De Roeck[1,2,†], Tobi Van den Bossche[1,2,3,4,†], Julie van der Zee[1,2], Jan Verheijen[1,2], Wouter De Coster[1,2], Jasper Van Dongen[1,2], Lubina Dillen[1,2], Yalda Baradaran-Heravi[1,2], Bavo Heeman[1,2], Raquel Sanchez-Valle[5], Albert Lladó[5], Benedetta Nacmias[6], Sandro Sorbi[6,7], Ellen Gelpi[8], Oriol Grau-Rivera[8], Estrella Gómez-Tortosa[9], Pau Pastor[10,11], Sara Ortega-Cubero[11], Maria A. Pastor[11,12,13], Caroline Graff[14,15], Håkan Thonberg[14,15], Luisa Benussi[16], Roberta Ghidoni[16], Giuliano Binetti[16,17], Alexandre de Mendonça[18], Madalena Martins[18], Barbara Borroni[19], Alessandro Padovani[19], Maria Rosário Almeida[20], Isabel Santana[20], Janine Diehl-Schmid[21], Panagiotis Alexopoulos[21], Jordi Clarimon[11,22], Alberto Lleó[11,22], Juan Fortea[11,22], Magda Tsolaki[23], Maria Koutroumani[24], Radoslav Matěj[25,26], Zdenek Rohan[25,26,27], Peter P. De Deyn[2,4], Sebastiaan Engelborghs[2,4], Patrick Cras[2,3], Christine Van Broeckhoven[1,2,*], Kristel Sleegers[1,2,*], on behalf of the European Early-Onset Dementia (EU EOD) consortium.

[1]Neurodegenerative Brain Diseases group, VIB Center for Molecular Neurology, Antwerp, Belgium

[2]Institute Born-Bunge, University of Antwerp, Antwerp, Belgium

[3]Department of Neurology, Antwerp University Hospital, Edegem, Belgium

[4]Department of Neurology and Memory Clinic, Hospital Network Antwerp (ZNA) Middelheim and Hoge Beuken, Antwerp, Belgium.

[5]Alzheimer's Disease and Other Cognitive Disorders Unit, Neurology Department, Hospital Clínic, Institut d'Investigacions Biomediques August Pi i Sunyer (IDIBAPS), Barcelona, Spain.

[6]Department of Neuroscience, Psychology, Drug Research and Child Health (NEUROFARBA), University of Florence, Florence, Italy.

[7]IRCCS Don Gnocchi, Florence, Italy

[8]Neurological Tissue Bank of the Biobanc, Hospital Clinic, Institut d'Investigacions Biomediques August Pi i Sunyer (IDIBAPS), Barcelona, Spain

[9]Department of Neurology, Fundación Jiménez Díaz, Madrid, Spain.

[10]Memory Unit. Department of Neurology, University Hospital Mútua de Terrassa, University of Barcelona School of Medicine, Terrassa, Barcelona, Spain.

[11]Centro de Investigación Biomédica en Red de Enfermedades Neurodegenerativas (CIBERNED), Instituto de Salud Carlos III, Madrid, Spain.

[12]Neuroimaging Laboratory, Division of Neurosciences, Center for Applied Medical Research (CIMA), University of Navarra, Pamplona, Spain.

[13]Department of Neurology, Clínica Universidad de Navarra, University of Navarra School of Medicine, Pamplona, Spain.

[14]Department of Neurobiology, Care Sciences and Society (NVS), Center for Alzheimer Research, Division of Neurogeriatrics, Karolinska Institutet, Huddinge, Sweden.

[15]Department of Geriatric Medicine, Genetics Unit, Karolinska University Hospital, Stockholm, Sweden.

[16]Molecular Markers Laboratory, Istituto di Ricovero e Cura a Carattere Scientifico (IRCCS), Istituto Centro San Giovanni di Dio-Fatebenefratelli, Brescia, Italy.

[17]MAC Memory Center, Istituto di Ricovero e Cura a Carattere Scientifico (IRCCS), Istituto Centro San Giovanni di Dio-Fatebenefratelli, Brescia, Italy

[18]Faculty of Medicine, University of Lisbon, Lisbon, Portugal.

[19]Centre for Neurodegenerative Disorders, Neurology Unit, Department of Clinical and Experimental Sciences, University of Brescia, Brescia

[20]Center for Neuroscience and Cell Biology, University of Coimbra, Coimbra, Portugal.

[21]Department of Psychiatry and Psychotherapy, Technische Universität München, München, Germany.

[22]Department of Neurology, IIB Sant Pau, Hospital de la Santa Creu i Sant Pau, Universitat Autònoma de Barcelona, Barcelona, Spain.

[23]3rd Department of Neurology, Medical School, Aristotle University of Thessaloniki, Makedonia, Greece.

[24]Laboratory of Biochemistry, Department of Chemistry, Aristotle University of Thessaloniki, Thessaloniki, Greece.

[25]Department of Pathology, First Medical Faculty, Charles University in Prague, Czech Republic.

[26]Department of Pathology and Molecular Medicine, Thomayer Hospital, Prague, Czech Republic.

[27]Institute of Pathology, Third Medical Faculty of Charles University in Prague, Prague, Czech Republic.

[†]These authors contributed equally

* Corresponding authors:

Prof. Dr. Kristel Sleegers MD PhD

Neurodegenerative Brain Diseases Group

VIB Center for Molecular Neurology, University of Antwerp - CDE

Universiteitsplein 1, B-2610, Antwerp, Belgium

Email: kristel.sleegers@molgen.vib-ua.be

Prof. Dr. Christine van Broeckhoven PhD DSc

Neurodegenerative Brain Diseases Group

VIB Center for Molecular Neurology, University of Antwerp - CDE

Universiteitsplein 1, B-2610, Antwerp, Belgium

Email: christine.vanbroeckhoven@molgen.vib-ua.be

Table of contents

Table of contents 4

Supplemental texts 5

Neuropathological description S1: neuropathological examination in an ABCA7 PTC carrier 5

Method S1: Western blotting 6

Supplemental tables 7

Table S1: Cohort characteristics for each country of origin 7

Table S2: MinION ABCA7 cDNA sequencing experimental setup. 8

Table S3: Characteristics of carriers of ABCA7 PTC mutations 9

Table S4: Haplotype of shared ABCA7 PTC mutation carriers 11

Table S5: Carriers of ABCA7 c.5570+5G>C in the EU EOD consortium 13

Table S6: Carriers of ABCA7 missense mutations with predicted deleterious effects (CADD > 20) 14

Table S7: Meta-analysis of association between common coding ABCA7 SNPs and EOAD in the EU EOD consortium. 16

Supplemental figures 17

Figure S1: Genotype phasing of two PTC mutations in one individual. 17

Figure S2: Dot plot showing the onset ages of ABCA7 PTC carriers versus carriers of a pathogenic PSEN1, PSEN2 or APP mutation. 18

Figure S3: MinION and Sanger cDNA sequencing of ABCA7 p.Met370fs. 19

Figure S4: MinION cDNA sequencing and Illumina RNA sequencing of ABCA7 p.Trp1336*. 20

Figure S5: MinION sequencing of ABCA7 p.Leu1403fs brain cDNA tissue. 22

Figure S6: Validation of ABCA7 exon 11 skipping transcripts with Illumina RNAseq. 23

Figure S7: Validation of ABCA7 exon 30 and 31 skipping with Illumina RNAseq. 24

Figure S8: MinION sequencing of ABCA7 p.Glu709fs. 25

Figure S9: Validation of alternative splicing of ABCA7 exon 16 with Illumina RNAseq. 26

Figure S10: MinION cDNA sequencing and Illumina RNA sequencing of ABCA7 c.5570+5G>C. 27

Figure S11: Hippocampal ABCA7 protein quantification in AD patients with or without an ABCA7 PTC mutation. 29

Supplemental references 30

Supplemental texts

Neuropathological description S1: neuropathological examination in an *ABCA7* PTC carrier

Neuropathological examination was performed in patient EOD-P1, showing high-level AD neuropathological changes (A3B3C3) as well as a cerebral amyloid angiopathy. The severe global cerebral atrophy was most pronounced in the anterior temporal lobes, with marked neuronal loss and gliosis in the neocortex (especially the frontal, temporal and parietal cortices) and limbic system. Amyloid pathology was frequent in the neocortex, hippocampus, amygdala, cingulum, striatum, and to a lesser extent in the thalamus, cerebellum and brainstem colliculi (CERAD neuritic plaque score of C and a Thal phase of 5, Figure 2a), in addition to abundant amyloid depositions in the wall of the cerebral and cerebellar leptomeningeal vessels (Figure 2b). Tau-positive neurofibrillary tangles and neuropil threads were frequent in the neocortex of the frontal, temporal, parietal and occipital lobes, as well as the cingulum, hippocampus and parahippocampal region (Figure 2c), amygdala, nucleus basalis of Meynert, and to a lesser extent in the brainstem neurons, especially those in the substantia nigra, periaqueductal gray matter, locus caeruleus and raphe nuclei. No α-synuclein immunoreactivity was found.

Method S1: Western blotting

Fresh frozen brain tissue was available for carriers of *ABCA7* PTC mutations c.67-1G>A and p.Leu1403fs. Hippocampus was extracted from these two carriers as well as from fresh frozen brain tissue from three AD patients not carrying an ABCA7 PTC mutation. To quantify ABCA7 expression with Western blotting, we adapted the protocol described by Allen and colleagues [1]. Protein lysates were prepared for Western blot using a 0.1% triton lysis buffer (150 mM NaCl, 50 mM Tris pH 7.5, 0.1% Triton). The protein concentration was determined with a BCA assay (Pierce, Rockford, IL, USA), and equal amounts of protein (80µg) were separated on an NuPAGE 3%–8% Tris-Acetate gel and transferred to a PVDF membrane (Hybond P, Amersham Biosciences, Little Chalfont, UK). Membranes were blocked in 5% milk in Phosphate-buffered saline with 0.1% Tween 20 (PBST) and probed overnight at 4°C with the ABCA7 primary antibody designed to epitope aa 2096-2146 (LS-C291064, LifeSpan BioSciences, Seattle, WA, USA; 1/500). Blots were incubated with rabbit IgG horseradish peroxidase–linked secondary antibody for 1 hour. Immunodetection was performed with the ECL-plus chemiluminescent detection system (Amersham Biosciences)). Equivalent sample loading was confirmed by probing with anti–glyceraldehyde 3-phosphate dehydrogenase (GAPDH) antibody (GTX100118, Genetex, Irvine, CA, USA; 1/20.000). Protein bands were quantified using ImageQuantTL software, and relative amounts of ABCA7 protein were determined. We observed lower protein expression in PTC mutation carriers in comparison to non-carrier patients (figure S11), confirming a reduction of expression by PTC mutations. Both PTC mutation carriers had no (c.67-1G>A), or low (4%; p.Leu1403fs) potential transcript rescue mechanisms. While a clear difference is shown between carriers and non-carriers, variability in expression is also observed reflective of external modifiers of ABCA7 expression. Of note, this antibody targets the C-terminus of ABCA7, and therefore measures full-length protein. Truncated proteins may be formed, but cannot be observed.

Supplemental tables

Table S1: Cohort characteristics for each country of origin

Country of origin	Patients (n=928)	Controls (n=980)
Spain	n=403<br>58.7% female<br>AAO = 57.8 ± 4.9 years<br>APOE ε4-positive = 52.5%	n=223<br>74.4% female<br>AAI = 58.6 ± 12.8 years<br>APOE ε4-positive = 16.4%
Italy	n=159<br>66.0% female<br>AAO = 55.9 ± 7.1 years<br>APOE ε4-positive = 38.4%	n=304<br>59.5% female<br>AAI = 65.3 ± 9.3 years<br>APOE ε4-positive = 23.0%
Sweden	n=160<br>63.1% female<br>AAO = 57.9 ± 4.6 years<br>APOE ε4-positive = 68.8%	n=295<br>61.0% female<br>AAI = 64.1 ± 5.4 years<br>APOE ε4-positive = 31.9%
Germany	n=83<br>51.8% female<br>AAO = 58.4 ± 4.7 years<br>APOE ε4-positive = 50.6%	n=0
Portugal	n=66<br>59.1% female<br>AAO = 56.3 ± 7.0 years<br>APOE ε4-positive = 42.4%	n=120<br>68.4% female<br>AAI = 66.3 ± 6.1<br>APOE ε4-positive = 24.2%
Greece	n=52<br>61.5% female<br>AAO = 57.5 ± 4.5 years<br>APOE ε4-positive = 46.2%	n=35<br>77.1% female<br>AAI = NA<br>APOE ε4-positive = 31.4%
Czech Republic	n=5<br>40.0% female<br>AAO = 52.3 ± 10.5 years<br>APOE ε4-positive = 60.0%	n=3<br>33.3% female<br>AAI = 56.3 ± 10.1 years<br>APOE ε4-positive = 33.3%

AAI: age at inclusion, AAO: age at onset, NA: not available

Table S2: MinION *ABCA7* cDNA sequencing experimental setup.

Mutation	Primers	RNA source	Read depth	cDNA amplicon size (bp)
c.67-1G>A	CGTTGTCCTGACCTCTCTGTC<br>GTCAGCTGCGGAAAGCAG	brain	1488	300
p.Met370fs	AACCGGACCTTCGAGGAG<br>TCAGGTCCCAAGAAGACGAC	blood	2311	398
p.Glu709fs	GCCTGGATCTACTCCGTGAC<br>AGCTCCTCCGAAAAGGAAAA	lymphoblast	4737	649
c.3577+1G>C	CTGCGGACACAGATATGGAG<br>AAAGAGGGCAGGCAGCAC	blood	1830	296
p.Trp1336*	TTTCTGTTGGTGCTGATATTGCCATGTACGGTGCTCAGGTGT<br>ACTTGCCTGTCGCTCTATCTTCGGCTGTGAGGTTTTTCAGGA	lymphoblast	3279	559
p.Leu1403fs	TTTCTGTTGGTGCTGATATTGCCATGTACGGTGCTCAGGTGT<br>ACTTGCCTGTCGCTCTATCTTCGGCTGTGAGGTTTTTCAGGA	brain	3477	559
c.5570+5G>C	TGTTGGTGCTGAGGAACTTG<br>GTTTGTTCCCTCCGCTGTAG	lymphoblast	3854	420

PTC mutations studied on transcript level are denoted by their HGVS notation. Forward and reverse primers were used to amplify the cDNA region of interest. Mutations p.Trp1336* and p.Leu1403fs are in close proximity and the same, but barcoded PCR amplicon, was used. RNA source corresponds to the mutation carrying patient biomaterial. Read depth is the number of reads obtained at the mutation of interest. The amplicon size is denoted according to the canonical *ABCA7* transcript (NM_019112) and may change due to alternative splicing.

Table S3: Characteristics of carriers of *ABCA7* PTC mutations

ABCA7 PTC mutation	ID	Origin	Gender	Diagnosis	Presenting features	APOE	Age	AAD	DD	FH	Other mutation
c.67-1G>A	EOD-P1	Spanish	f	definite AD[§]	-	34	≤59	68	9	F	-
	EOD-P2	Spanish	f	probable AD	amnestic	33	58	-	-	F	-
	EOD-P3	Spanish	f	probable AD	amnestic	33	64	-	-	F	-
p.His44fs	EOD-P4	Swedish	f	probable AD	amnestic	23	54	66	12	S	-
p.Trp69*<br>c.579+1G>T	EOD-P5[†]	Portuguese	m	probable AD	amnestic	44	52	-	-	S	-
c.302+1G>C	EOD-P6.1[φ]	Spanish	m	probable AD	amnestic + behavioral	34	42	49	7	F	PSEN1 - p.H163R
p.Met370fs	EOD-P7	Spanish	f	probable AD	amnestic	43	52	-	-	S	-
p.Cys659fs	EOD-P8	Italian	f	probable AD	-	33	49	-	-	S	-
p.Glu709fs	EOD-P9	Italian	m	probable AD	amnestic	33	58	-	-	S	-
	EOD-P10	Spanish	f	probable AD	amnestic + language	33	55	62	7	S	-
	EOD-P11	Swedish	f	probable AD	amnestic	34	59	-	-	-	-
	EOD-P12	Spanish	f	probable AD	amnestic	43	60	-	-	F	-
	EOD-P13	Spanish	f	probable AD	amnestic	33	62	-	-	S	-
	EOD-P14	Spanish	f	probable AD	amnestic	33	60	70	10	F	-
p.Glu712*	EOD-P15	Greek	f	AD	-	33	55	-	-	-	-
p.Gln732*	EOD-P16	Portuguese	m	probable AD	amnestic + dysexecutive	33	50	57	7	F	-
p.Thr849fs	EOD-P17	Portuguese	f	probable AD	amnestic	33	59	-	-	F	-
	EOD-P18	Portuguese	f	probable AD	amnestic	33	65	-	-	F	-
c.3577+1G>C	EOD-P19	Spanish	f	probable AD	logopenic PPA	44	61	68	7	F	SORL1 - p.H1813R[Φ]
p.Leu1403fs	EOD-P20	Spanish	f	probable AD	amnestic	34	48	58	10	S	-
	EOD-P21.1	Italian	f	probable AD	-	34	60	-	-	F	-
	EOD-P22	Spanish	f	probable AD	amnestic	44	65	-	-	F	-
	EOD-P23	German	f	probable AD	-	44	63	-	-	F	-
	EOD-P24	Spanish	f	AD	amnestic – slow evolution	34	60	-	-	F	-
p.Trp1461*	EOD-P25	Italian	m	probable AD	amnestic + language + dysexecutive	44	57	-	-	F	-
p.Arg1489*	EOD-P26	Spanish	m	probable AD	amnestic	33	55	-	-	F	-
	EOD-P27	Portuguese	f	probable AD	amnestic	33	55	-	-	S	-
	EOD-P28	Spanish	m	AD vs FTD	amnestic	33	50	-	-	S	-
p.Glu709fs	EOD-C1	Italian	m	CON	-	23	67	-	-	-	-
	EOD-C2	Swedish	f	CON	-	23	60	-	-	-	-
p.Trp1336*	EOD-C3	Italian	m	CON	-	34	58	-	-	-	-

p.Leu1403fs	EOD-C4	Italian	f	CON	-	33	44	-	-	-	-
c.4416+2T>G	EOD-C5	Italian	m	CON	-	33	72	-	-	-	-
	EOD-C6	Italian	f	CON	-	33	73	-	-	-	-

PTC mutations validated in early onset patients (AD) and control individuals (CON). AAD = age at death, AD = Alzheimer's disease, CON = control, DD = disease duration, FH = familial history, F = familial, PPA = primary progressive aphasia S = sporadic. Age refers to the onset age for patients and inclusion age for controls. Mutation nomenclature is provided according to Human Genome Variation Society (HGVS) on either the transcript or protein (if applicable) level. † This individual carried 2 PTC mutations that segregated on the same haplotype (see Figure S1). § For a description, see Neuropathological description S1. ϕ This *SORL1* missense mutation of unknown importance was previously observed [3]. φ Note: EOD-P6.1 was not included in genetic association testing due to a pathogenic *PSEN1* mutation.

Table S4: Haplotype of shared *ABCA7* PTC mutation carriers

Marker	Genomic location	Distance to ABCA7 (kb)	Heterozygosity/MAF	p.Glu709fs	p.Leu1403fs
D19S814	chr19:599821-600026	-440	NA	-	-
D19S886	chr19:998643-998960	-41	0.63	210*	212*
rs3764645	chr19:1042809	0	0.4	G	G
rs3764648	chr19:1044753	0	0.283	C	C
rs3752234	chr19:1047002	0	0.421	A	G
rs3752237	chr19:1047161	0	0.305	G	G
rs4147913	chr19:1049165	0	0.407	C	C
rs3752240	chr19:1051214	0	0.289	A	G
rs3764651	chr19:1051751	0	0.49	G	A
rs3764652	chr19:1052005	0	0.379	T	C
rs3829687	chr19:1053299	0	0.477	T	C
rs3752242	chr19:1053677	0	0.382	A	G
rs3752243	chr19:1054060	0	0.464	G	A
rs3745842	chr19:1055191	0	0.391	A	G
rs881768	chr19:1056065	0	0.442	G	A
rs2279796	chr19:1059004	0	0.425	G	A
rs34606911	chr19:1062370	0	0.233	T	C
rs4807499	chr19:1063930	0	0.202	C	T
rs4147930	chr19:1064193	0	0.394	G	A
rs2242437	chr19:1065563	0	0.395	G	C
D19S883	chr19:1413756-1414123	348	0.73	-	-
D19S878	chr19:2359696-2359954	1294	0.82	-	-
D19S424	chr19:3226372-3226704	2161	0.79	-	-
D19S894	chr19:4392405-4392667	3327	0.78	-	-
D19S177	chr19:5517296-5517670	4452	0.76	-	-
**Number of variant carriers**				8	6

For individuals sharing the same PTC mutations we determined haplotypes based on SNPs and STR markers spanning *ABCA7* and flanking regions as previously described [2]. Briefly, we selected 18 common SNPs (rs-numbers) that were covered within our targeting assay while passing Hardy Weinberg Equilibrium quality control (p>0.001). In addition, seven STR markers (D19S-notation) were genotyped with FAM-labeled multiplex PCR, supplemented with GeneScan™ 500 LIZ™ size standard (Thermo Fisher Scientific, Waltham, USA) after which capillary fragment analysis was performed on an ABI 3730 DNA Analyzer (Thermo Fisher Scientific, Waltham, USA). Heterozygosities of STR markers were obtained from

the Marshfield Clinic database (www.marshfieldclinic.org) and MAF of SNPs was based on 1000 genomes data. *ABCA7* is located in the 19p subtelomeric region, hence only two STR markers were selected on the telomeric side of *ABCA7*. The shared haplotype is shown per PTC variant. '-' = Samples did not share this marker. '*'This STR allele was absent in 1 individual carrying the respective mutation.

Table S5: Carriers of *ABCA7* c.5570+5G>C in the EU EOD consortium

Sample ID	Status	Gender	Origin	APOE	Age	Family History	c.5570+5G>C zygosity
EOD-P45	AD	m	Italian	33	51	S	Heterozygous
EOD-P46	AD	m	Spanish	34	53	S	Heterozygous
EOD-P47	AD	m	Spanish	44	61	F	Heterozygous
EOD-P48	AD	m	Swedish	33	56	-	Homozygous
EOD-P49	AD	f	Swedish	33	56	-	Heterozygous
EOD-P50	AD	f	Swedish	33	64	-	Heterozygous
EOD-C16	CON	f	Italian	33	-	-	Heterozygous
EOD-C17	CON	-	Portuguese	33	-	-	Heterozygous
EOD-C18	CON	m	Spanish	34	-	-	Heterozygous
EOD-C19	CON	f	Swedish	33	61	-	Heterozygous
EOD-C20	CON	f	Swedish	23	61	-	Heterozygous
EOD-C21	CON	f	Swedish	33	66	-	Heterozygous
EOD-C15	CON	f	Swedish	33	60	-	Heterozygous

Age = onset age for patients (AD) and inclusion age for controls (CON). F = positive familial history (affected first degree relative). S = sporadic. Heterozygous = GC genotype, Homozygous = CC genotype.

Table S6: Carriers of *ABCA7* missense mutations with predicted deleterious effects (CADD > 20)

Mutation coordinates and nomenclature			Effect prediction			Carrier characteristics						
Genomic position	HGVS (coding)	HGVS (protein)	PolyPhen category	SIFT category	Phred CADD score	Sample ID	Status	Gender	Origin	APOE	Age	FH
chr19:1045025	c.1240G>A	p.Ala414Thr	benign	deleterious	24.1	EOD-P30	AD	m	German	33	59	-
chr19:1046288	c.1505G>C	p.Gly502Ala	possibly damaging	deleterious	25	EOD-C14	CON	m	Swedish	33	60	-
chr19:1047168	c.1858C>T	p.Leu620Phe	probably damaging	deleterious	31	EOD-P32	AD	f	Greek	34	60	-
chr19:1047168	c.1858C>T	p.Leu620Phe	probably damaging	deleterious	31	EOD-P36	AD	f	Italian	34	58	F
chr19:1047169	c.1859T>C	p.Leu620Pro	probably damaging	deleterious	28.6	EOD-P41	AD	f	Spanish	44	57	F
chr19:1047336	c.2026G>A	p.Ala676Thr	probably damaging	deleterious	27.1	EOD-P40†	AD	f	Spanish	33	58	F
chr19:1047336	c.2026G>A	p.Ala676Thr	probably damaging	deleterious	27.1	EOD-C10†	CON	m	Portuguese	33	-	-
chr19:1047336	c.2026G>A	p.Ala676Thr	probably damaging	deleterious	27.1	EOD-C11†	CON	f	Spanish	33	-	-
chr19:1047498	c.2114C>A	p.Ala705Asp	probably damaging	deleterious	28.9	EOD-P37	AD	f	Spanish	33	56	S
chr19:1049283	c.2399C>T	p.Pro800Leu	probably damaging	tolerated	24.7	EOD-P31	AD	f	Greek	33	49	-
chr19:1051006	c.2639G>A	p.Arg880Gln	probably damaging	deleterious	34	EOD-P29	AD	f	German	44	58	-
chr19:1051006	c.2639G>A	p.Arg880Gln	probably damaging	deleterious	34	EOD-P44	AD	m	Swedish	34	59	-
chr19:1051006	c.2639G>A	p.Arg880Gln	probably damaging	deleterious	34	EOD-C13	CON	f	Swedish	34	61	-
chr19:1051006	c.2639G>A	p.Arg880Gln	probably damaging	deleterious	34	EOD-C15	CON	f	Swedish	33	60	-
chr19:1051944	c.2966G>A	p.Arg989His	probably damaging	deleterious	33	EOD-C12	CON	f	Spanish	33	-	-
chr19:1051964	c.2986C>T	p.His996Tyr	probably damaging	deleterious	28.1	EOD-C7	CON	m	Italian	33	-	-
chr19:1051964	c.2986C>T	p.His996Tyr	probably damaging	deleterious	28.1	EOD-C8	CON	m	Italian	33	-	-
chr19:1052067	c.3089G>T	p.Gly1030Val	possibly damaging	deleterious	26.9	EOD-P39	AD	f	Spanish	34	65	S

chr19:1053504	c.3397G>A	p.Gly1133Arg	probably damaging	deleterious	34	EOD-P34	AD	f	Italian	34	62	F
chr19:1057919	c.4886C>T	p.Ser1629Leu	probably damaging	deleterious	35	EOD-P38	AD	f	Spanish	33	50	S
chr19:1057949	c.4922_4924delTCT	p.Phe1641del	NA	NA	20.7	EOD-P40†	AD	f	Spanish	33	58	F
chr19:1058635	c.5168C>T	p.Ser1723Leu	probably damaging	deleterious	33	EOD-P40†	AD	f	Spanish	33	58	F
chr19:1058635	c.5168C>T	p.Ser1723Leu	probably damaging	deleterious	33	EOD-P42	AD	f	Spanish	33	47	-
chr19:1058635	c.5168C>T	p.Ser1723Leu	probably damaging	deleterious	33	EOD-C10†	CON	m	Portuguese	33	-	-
chr19:1058635	c.5168C>T	p.Ser1723Leu	probably damaging	deleterious	33	EOD-C11†	CON	f	Spanish	33	-	-
chr19:1058673	c.5206G>T	p.Ala1736Ser	possibly damaging	deleterious	25.7	EOD-P33	AD	f	Italian	33	62	-
chr19:1058939	c.5400G>C	p.Lys1800Asn	probably damaging	deleterious	28.5	EOD-C9	CON	m	Italian	33	46	-
chr19:1059079	c.5458G>A	p.Gly1820Ser	probably damaging	deleterious	32	EOD-P43	AD	m	Swedish	33	64	-
chr19:1061817	c.5500A>G	p.Thr1834Ala	possibly damaging	deleterious	26	EOD-P35	AD	f	Italian	33	47	S

Individuals carrying a missense mutation with a Phred-scaled CADD score higher than 20 are shown. In addition, predicted effects of PolyPhen and Sift (incorporated into CADD) are shown. Age = onset age for patients (AD) and inclusion age for controls (CON). FH = Familial History. F = positive familial history (affected first degree relative). S = sporadic. † These individuals carried a “double deleterious missense” haplotype.

Table S7: Meta-analysis of association between common coding *ABCA7* SNPs and EOAD in the EU EOD consortium.

Mutation coordinates and nomenclature					MAF		Association	
**genomic position (hg19)**	**HGVS (coding)**	**HGVS (protein)**	**dbSNP**	**MA**	**AD (%)**	**CON (%)**	**OR (95% CI)**	**p-value**
chr19:1043103	c.G643A	p.G215S	rs72973581	A	3.33	4.74	0.60 (0.42 - 0.87)	0.006
chr19:1061804	c.T5487C	p.N1829N	rs78320196	C	3.74	5.68	0.65 (0.47 - 0.90)	0.009
chr19:1042809	c.A563G	p.E188G	rs3764645	G	41.40	45.32	0.86 (0.75 - 0.99)	0.030
chr19:1064193	c.G5985A	p.L1995L	rs4147930	G	27.93	30.04	0.89 (0.76 - 1.03)	0.121
chr19:1053524	c.C3417G	p.L1139L	rs3752241	G	14.71	15.83	0.88 (0.72 - 1.07)	0.193
chr19:1051214	c.A2745G	p.V915V	rs3752240	G	32.78	35.28	0.91 (0.79 - 1.06)	0.218
chr19:1043748	c.A955G	p.T319A	rs3752232	G	2.56	3.17	0.77 (0.50 - 1.20)	0.256
chr19:1047537	c.A2153C	p.N718T	rs3752239	C	2.56	3.23	0.84 (0.55 - 1.28)	0.424
chr19:1041852	c.G183T	p.L61L	rs3764644	T	2.76	3.31	0.86 (0.57 - 1.29)	0.459
chr19:1054060	c.A3528G	p.L1176L	rs3752243	G	43.16	41.79	1.05 (0.92 - 1.21)	0.452
chr19:1052005	c.C3027T	p.A1009A	rs3764652	T	43.43	42.18	1.05 (0.92 - 1.21)	0.458
chr19:1062164	c.5571-7T>C	-	rs4147920	C	2.60	3.18	0.88 (0.57 - 1.35)	0.566
chr19:1062192	c.T5592C	p.A1864A	rs4147921	C	2.55	3.07	0.88 (0.57 - 1.36)	0.571
chr19:1056065	c.A4239G	p.R1413R	rs881768	G	43.08	41.98	1.05 (0.91 - 1.20)	0.516
chr19:1058176	c.A5057G	p.Q1686R	rs4147918	G	2.71	3.42	0.89 (0.59 - 1.34)	0.582
chr19:1044712	c.A1184G	p.H395R	rs3764647	G	2.98	3.72	0.89 (0.58 - 1.36)	0.586
chr19:1055191	c.G4046A	p.R1349Q	rs3745842	A	42.52	41.45	1.03 (0.90 - 1.19)	0.652
chr19:1047161	c.A1851G	p.G617G	rs3752237	A	41.50	40.48	1.02 (0.88 - 1.19)	0.768
chr19:1056492	c.G4580C	p.G1527A	rs3752246	G	21.48	20.52	1.02 (0.86 - 1.20)	0.850
chr19:1049269	c.G2385A	p.L795L	rs4147914	A	16.18	16.60	0.98 (0.81 - 1.17)	0.799
chr19:1049305	c.C2421A	p.V807V	rs4147915	A	12.72	12.60	1.02 (0.83 - 1.26)	0.823
chr19:1047002	c.A1824G	p.A608A	rs3752234	A	46.68	46.37	0.99 (0.86 - 1.14)	0.884

Genomic coordinates are based on hg19. Fixed effects (Cochran-Mantel-Haenszel) meta-analysis was performed on all common (minor allele frequency (MAF) > 1%) coding SNPs. HGVS = mutation nomenclature according to the Human Genome Variation Society. dbSNP notations refer to Reference SNP IDs (rs) from dbSNP build 142. MA = minor allele. AD = Alzheimer's Disease. CON = Control individual. Odds ratios (OR) and 95% confidence intervals (CI) are calculated for the MA. The study-wide multiple testing corrected p-value cutoff is 0.0033.

Supplemental figures

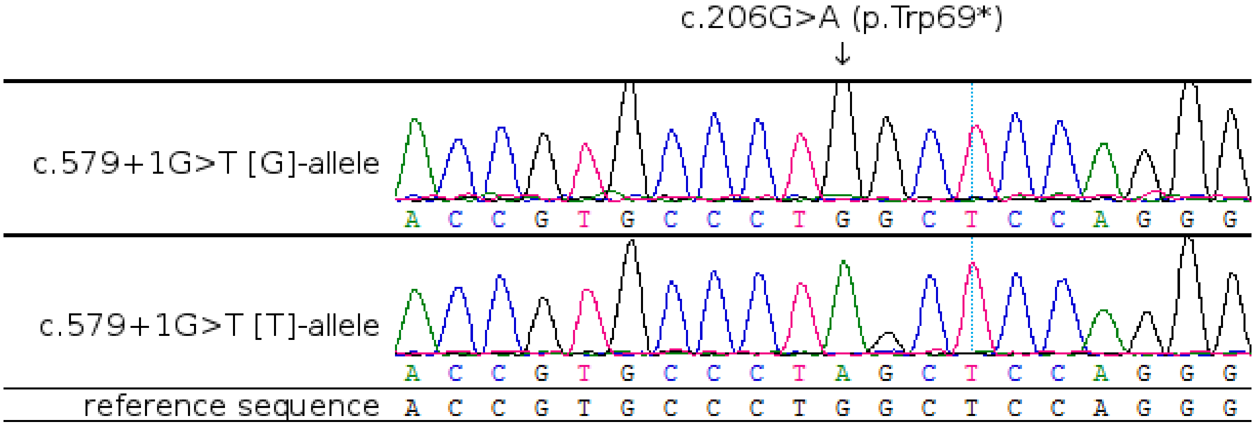

Figure S1: Genotype phasing of two PTC mutations in one individual. One patient (EOD-P5) carried two PTC mutations (c.206G>A (p.Trp69*) and c.579+1G>T). Allele-specific PCR was performed to resolve haplotype phase. The final 3’ nucleotide of the reverse primer corresponded with either the G (5’-GGATGAGTGGGGCCTCGTAC-3’) or T (5’-GGATGAGTGGGGCCTCGTAA-3’) allele of variant c.579+1G>T. PCR amplification of the fragment containing c.206G>A was separately performed for each c.579+1G>T allele in combination with a common forward primer (5’-CAGGGACCAGGCACTTTGTG-3’), after which Sanger sequencing was performed with the forward primer. Both mutations segregated in cis as shown in the figure: The reference c.579+1G>T[G]-allele (upper chromatogram panel) carried the reference c.206G>A[G]-allele, while the PTC c.579+1G>T[T]-allele (second chromatogram panel) co-segregated with the PTC c.206G>A[A]-allele. The bottom nucleotide track corresponds to the reference sequence (hg19).

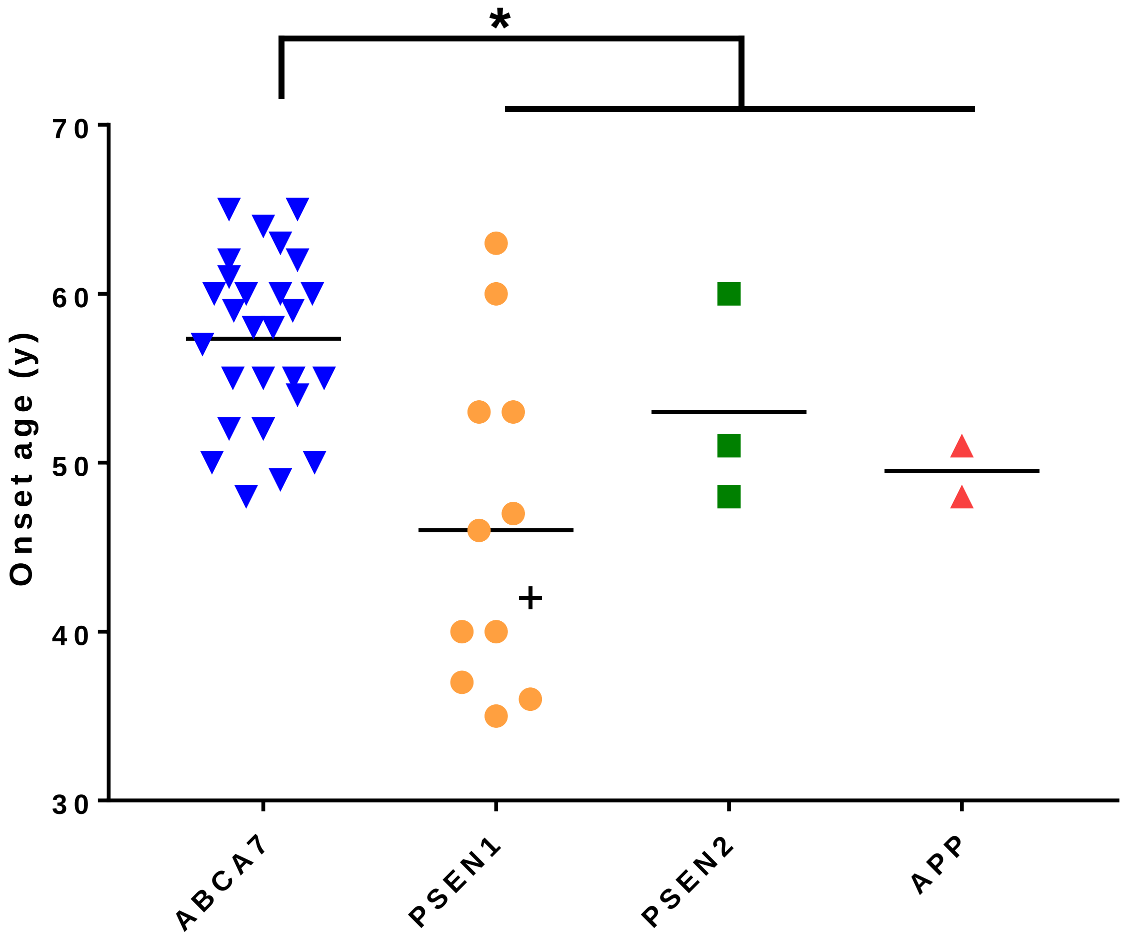

Figure S2: Dot plot showing the onset ages of *ABCA7* PTC carriers versus carriers of a pathogenic *PSEN1*, *PSEN2* or *APP* mutation. Mann-Whitney *U* test *p* value 0.0002. The cross denotes an individual who carries an *ABCA7* PTC mutation (c.302+1G>C) in addition to a pathogenic *PSEN1* p.His163Arg mutation. Note: all are EOAD patients, hence all onset ages are equal to or below 65 years.

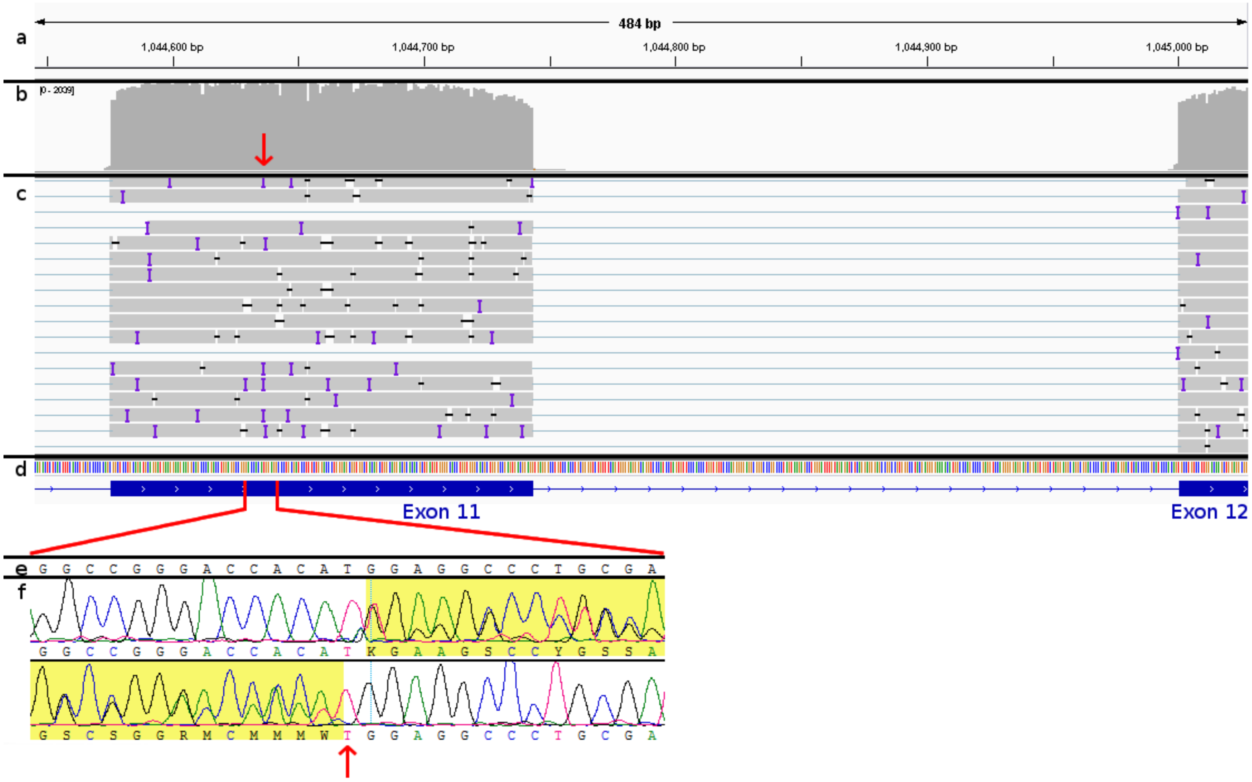

Figure S3: MinION and Sanger cDNA sequencing of *ABCA7* p.Met370fs. The cDNA sequencing shown here, was derived from blood RNA of a p.Met370fs (c.1109dupT) mutation carrier. The upper panels (a-d) correspond to IGV visualization of MinION sequencing data: **(a)** Coordinates on chromosome 19 (hg19) and location of the PTC mutation (red arrow), corresponding to features in panels b, c, and d. **(b)** Coverage plot in which the height of each bar corresponds to the read depth at that nucleotide position. Gray color indicates that the hg19 reference nucleotide was observed **(c)** A snapshot of sequencing reads. Each gray bar represents aligned sequences, which are connected by blue lines that correspond to splicing events. In this example, three reads show skipping of exon 11 (which contains p.Met370fs). **(d)** A color code corresponding to reference nucleotides is shown and below blue bars and lines depict *ABCA7* exons and introns, respectively. The red lines show the region that is represented in more detail below (e-f) using Sanger sequencing: **(e)** The reference sequence. **(f)** Forward (top) and reverse (bottom) Sanger sequencing of the c.1109dupT mutation (red arrow), which causes insertion of a T allele and hence a shift in nucleotides (yellow highlights).

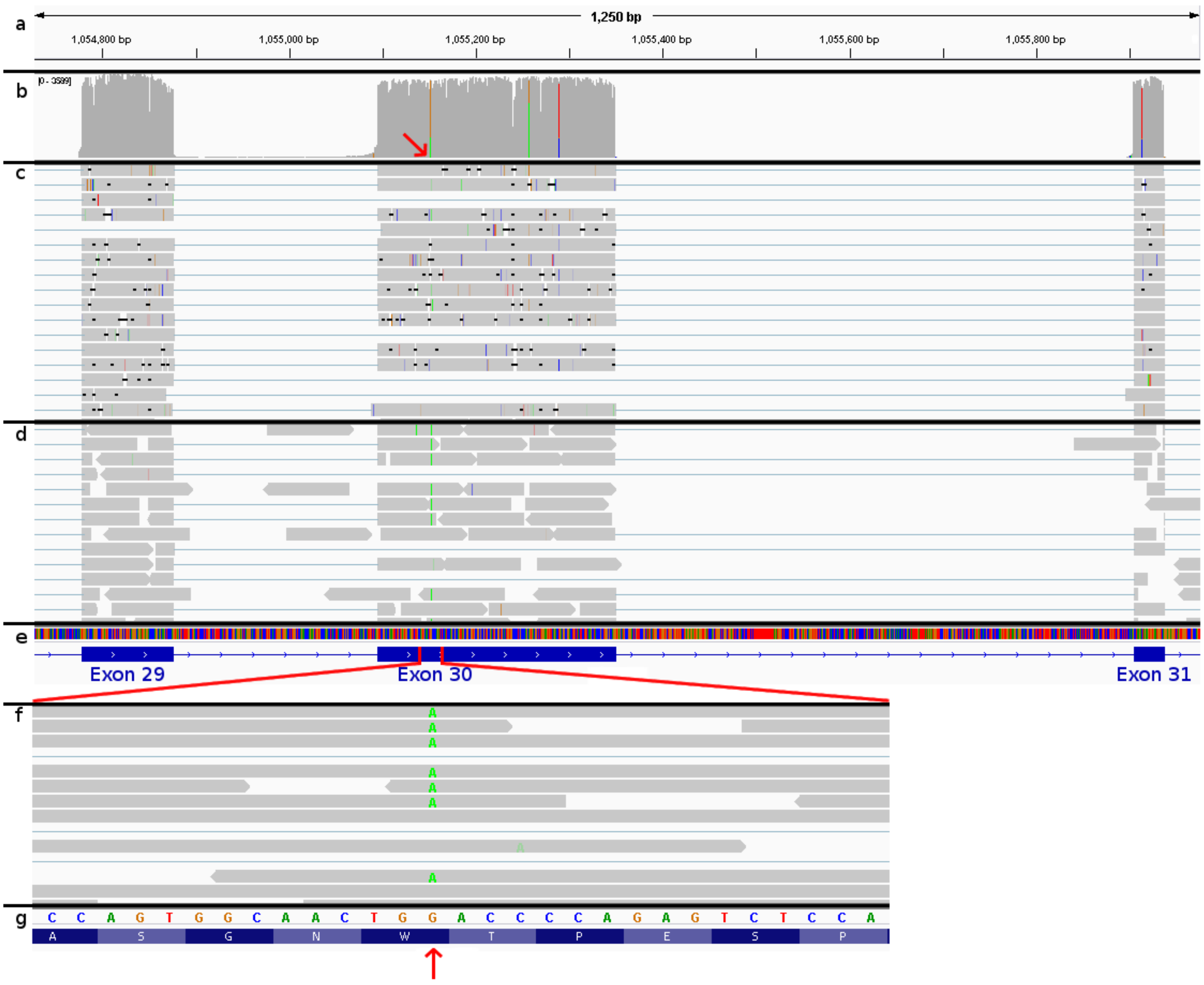

Figure S4: MinION cDNA sequencing and Illumina RNA sequencing of *ABCA7* p.Trp1336*.

RNA and cDNA were obtained from patient lymphoblasts. Sequencing reads were visualized with IGV. **(a)** Coordinates on chromosome 19 (hg19) and location of the PTC mutation (red arrow), corresponding to features in panels b, c, d, and e. **(b)** Coverage plot in which the height of each bar corresponds to the read depth at that nucleotide position. Colors are shown depending on the observed nucleotides, and gray color indicates that the hg19 reference nucleotide was observed. **(c)** This panel represents MinION cDNA sequencing. Gray bars correspond to aligned sequences and blue lines denote splicing events. Hence several canonically spliced transcripts are shown, as well as reads skipping exon 30 (4 reads in this figure). Due to long length sequencing on a MinION platform, each read shown here encompasses the entire selected part of the *ABCA7* transcript. The MinION produces a higher error rate than Illumina as depicted by the mismatches, deletions, and insertions shown in the aligned sequences. Accuracy, however, is high enough for alignment and calculation of splicing, and at high read depth, these errors are negated. **(d)** This panel shows RNAseq, validating the exon skipping shown in panel c. Read lengths (depicted by gray segments) produced by Illumina RNAseq are much shorter than those generated by MinION. Furthermore the number of reads in RNAseq aligning to *ABCA7* is very low due to low

expression of this gene. Even though the error rate is lower this compromises a semi-quantitative assessment of alternative splicing isoforms. **(e)** An overview of the nucleotides (color code track) and *ABCA7* gene layout (blue). The red lines denote a region that was analyzed at higher depth in panels f and g. **(f)** Zoomed in RNAseq reads clearly depict the presence of the nonsense p.Trp1336[A]-allele (red arrow), as well as exon 30 skipping. **(g)** The reference nucleotides and amino acids to which the reads in panel f align.

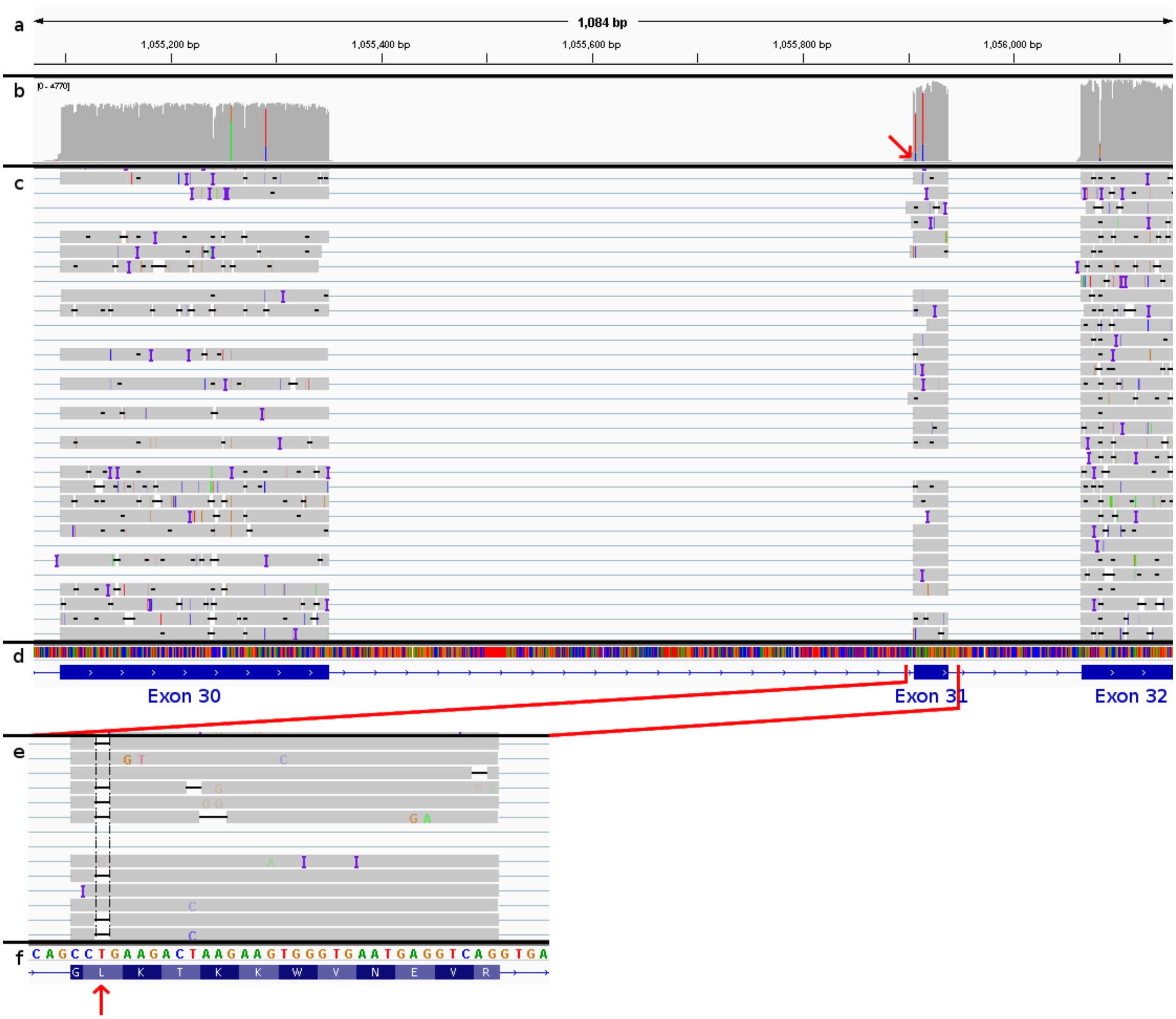

Figure S5: MinION sequencing of *ABCA7* p.Leu1403fs brain cDNA tissue. IGV visualization of long-read cDNA sequencing. **(a)** Coordinates on chromosome 19 (hg19) and location of the PTC mutation (red arrow), corresponding to features in panels b, c, and d. **(b)** Coverage plot in which the height of each bar corresponds to the read depth at that nucleotide position. Colors are shown depending on the observed nucleotides, and gray color indicates that the hg19 reference nucleotide was observed. **(c)** Each row represents aligned sequencing reads (gray bars) and splicing events (blue lines). Skipping of exon 31 harboring the p.Leu1403fs mutation is visible (4%). This visualization also illustrates relatively common skipping of exon 30 (30%). Of note, one of the other PTC mutations, p.Trp1336* (observed in a separate control individual – see Figure S4), is located in this exon. **(d)** An overview of the nucleotides (color code track) and *ABCA7* gene layout. The red lines denote PTC carrying exon 31, which is shown in more detail in panels e and f. **(e)** A snapshot of reads covering exon 31, which depicts the deletion (black horizontal lines) of a thymidine residue (red arrow) leading to p.Leu1403fs. **(f)** Reference nucleotides and amino acids in exon 31.

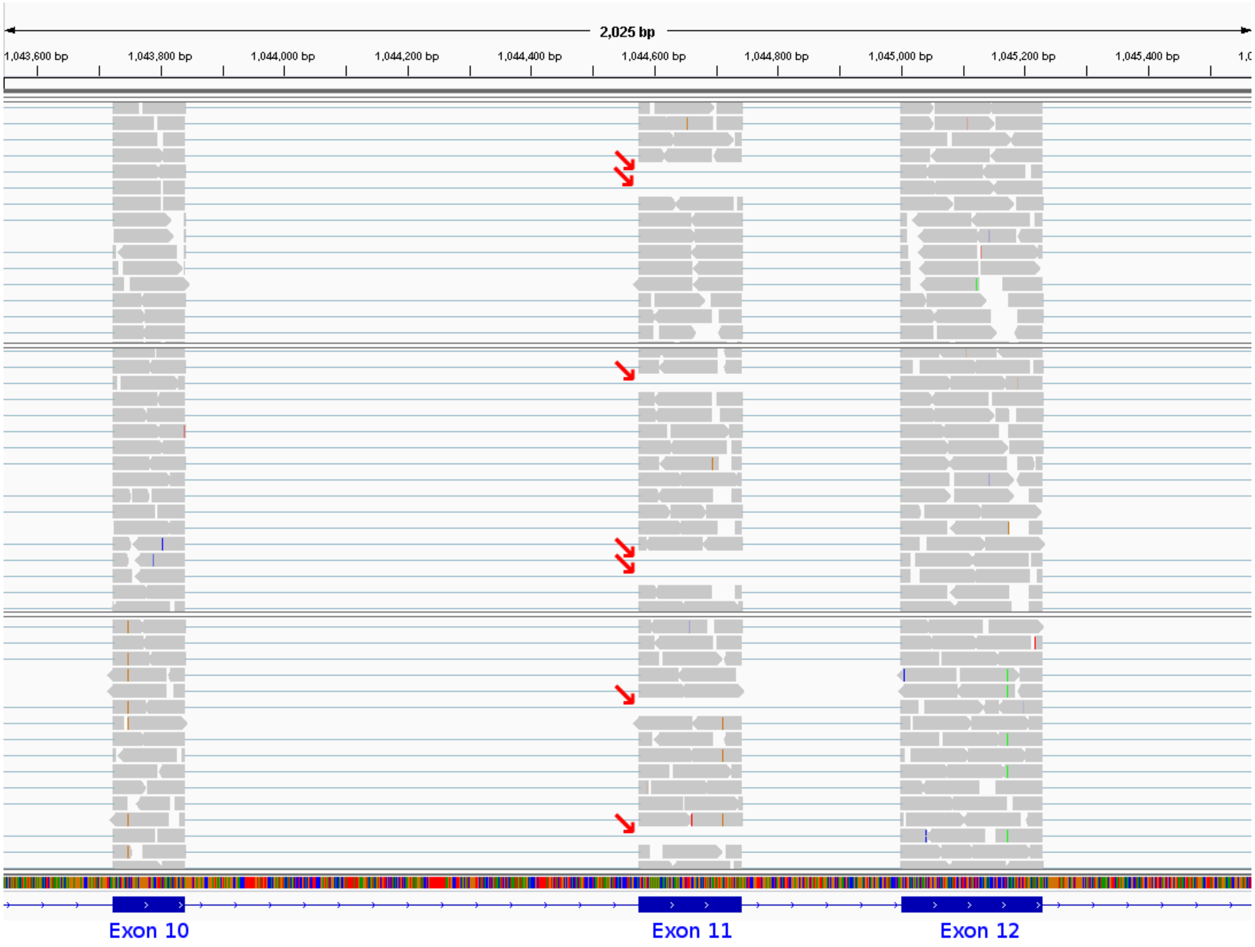

Figure S6: Validation of ABCA7 exon 11 skipping transcripts with Illumina RNAseq. IGV visualization of sequencing reads originating from lymphoblast RNA [3]. Coordinates on chromosome 19 (hg19) are shown on top. The three panels each correspond to a different individual and depict aligned sequencing reads (gray bars) as well as splicing events (blue connecting lines). At the bottom, a color code track corresponds to different nucleotides in the reference sequence and the canonical ABCA7 transcript (NM_019112) is shown in blue.

MinION sequencing (Figure S3) demonstrated inframe exon 11 skipping as a potential rescue mechanism for PTC mutations located in exon 11 (e.g. p.Met370fs). The RNAseq samples depicted here, do not carry a PTC mutation in exon 11, and show a common occurrence of this novel *ABCA7* isoform (red arrows). Exon 11 skipping is therefore not exclusive to exon 11 PTC mutation carriers, but can potentially rescue the negative effect of a PTC mutation in exon 11.

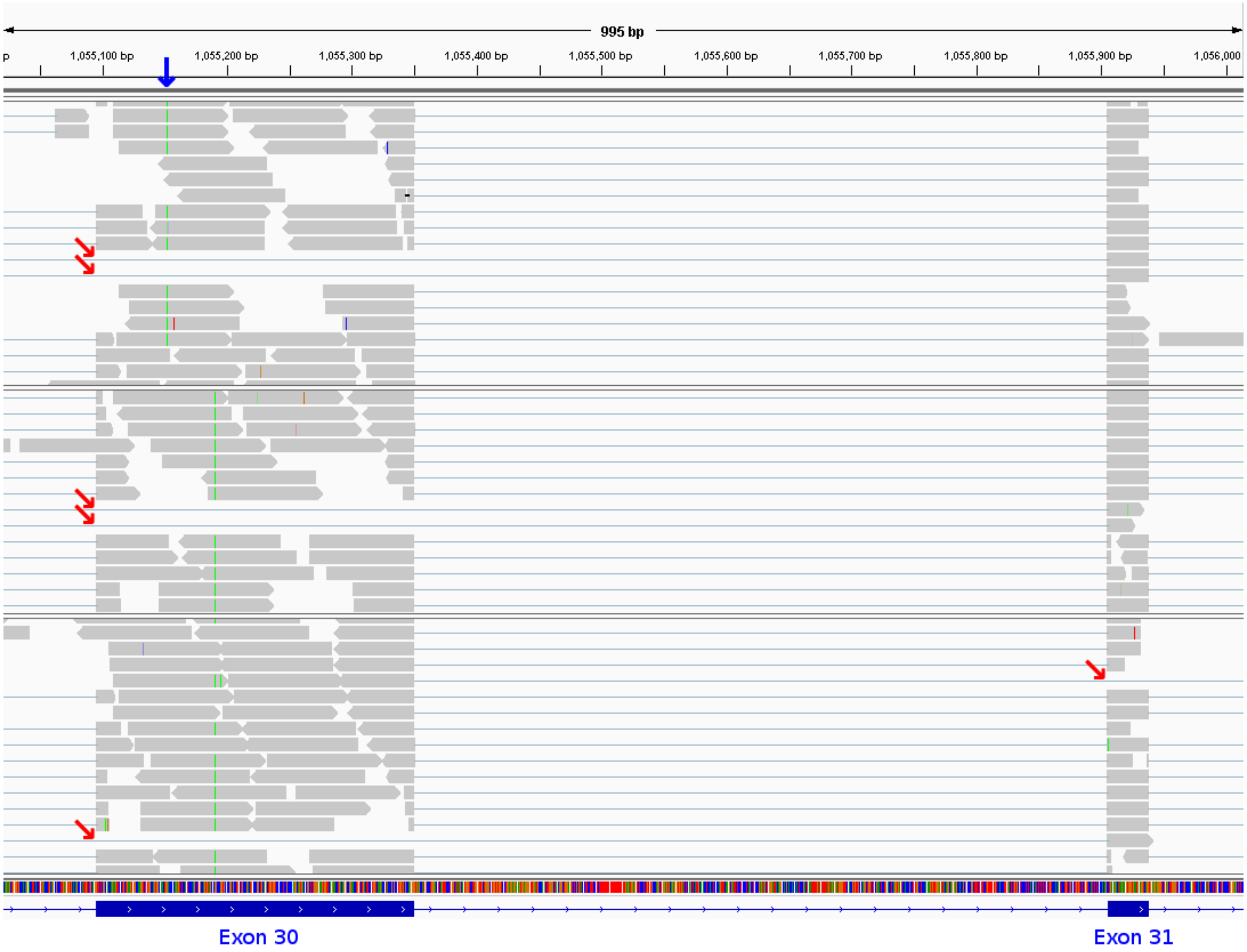

Figure S7: Validation of ABCA7 exon 30 and 31 skipping with Illumina RNAseq. IGV visualization of sequencing reads originating from lymphoblast RNA [3]. Coordinates on chromosome 19 (hg19) are shown on top. The three panels below each correspond to a different individual and depict aligned sequencing reads (gray bars) as well as splicing events (blue connecting lines). At the bottom, a color code track corresponds to different nucleotides in the reference sequence and the canonical ABCA7 transcript (NM_019112) is shown in blue.

Both exon 30 and 31 contain known PTC mutations (respectively p.Trp1336* and p.Leu1403fs). With MinION cDNA sequencing we observed inframe skipping of exon 30 and 31, which has the potential to diminish deleterious mutational effects. With RNAseq data we can validate both exon skipping events (red arrows). The upper panel corresponds to an p.Trp1336* carrier (location of the mutation is depicted with a blue arrow).

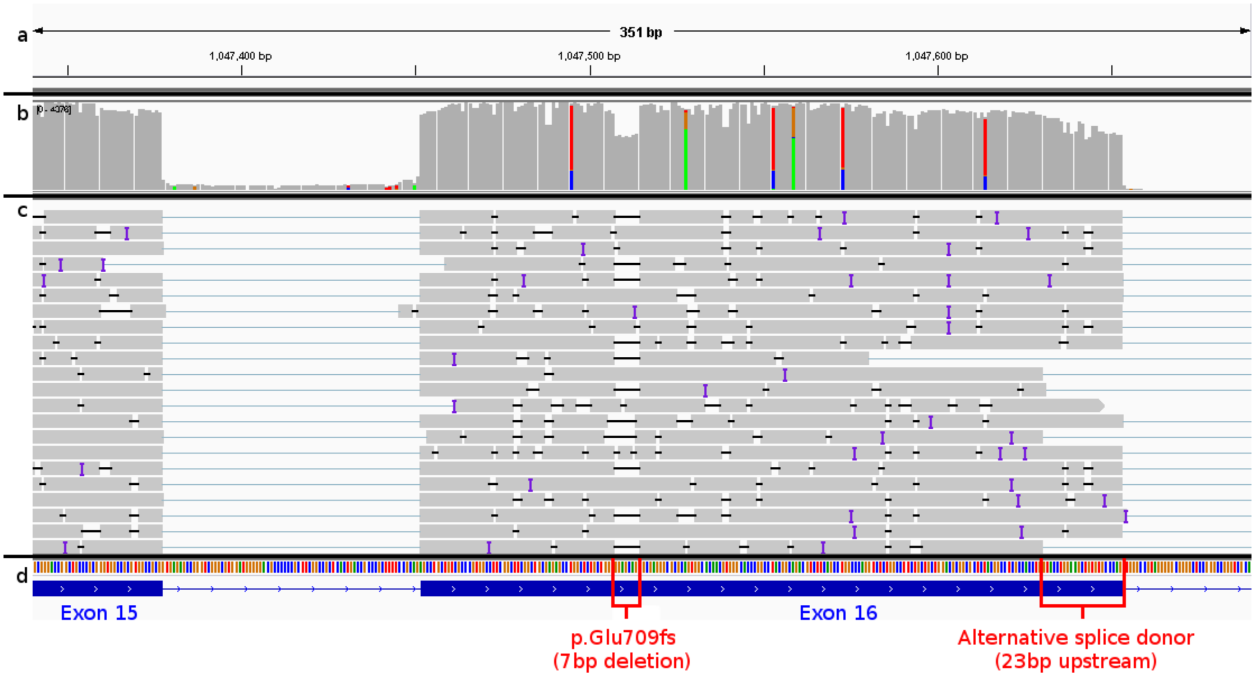

Figure S8: MinION sequencing of *ABCA7* p.Glu709fs. IGV visualization of sequencing reads from MinION cDNA sequencing on lymphoblast RNA of a p.Glu709fs carrying patient. **(a)** Coordinates on chromosome 19 (hg19), corresponding to features in panels b, c, and d. **(b)** Coverage plot in which the height of each bar corresponds to the read depth at that nucleotide position. Colors are shown depending on the observed nucleotides, and gray color indicates that the hg19 reference nucleotide was observed. **(c)** aligned sequencing reads (gray bars) and splicing events (blue lines). **(d)** An overview of the nucleotides (color code track) and *ABCA7* gene layout (blue) along with annotated events of interest (red).

The 7bp deletion (black horizontal lines in panel c, and drop in coverage in panel b) leading to p.Glu709fs is observed in *ABCA7* transcripts. While most reads follow a canonical splicing pattern, some p.Glu709fs transcripts are alternatively spliced by usage of a cryptic splice donor site 23bp upstream of the canonical splice donor site, with restoration of the reading frame as a consequence. Additionally other splicing events are observed as well (e.g. usage of a cryptic exon 16 splice donor site 73bp upstream).

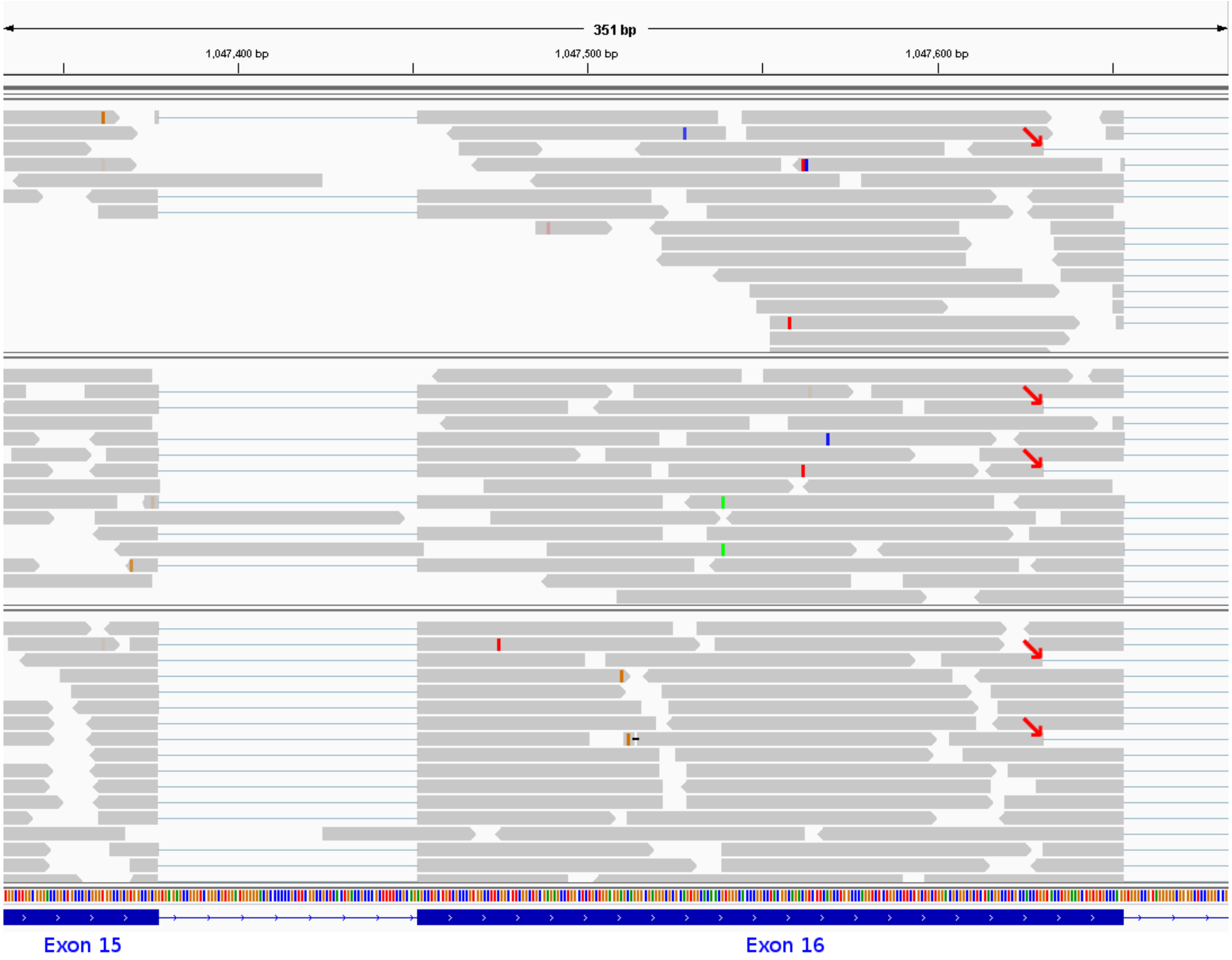

Figure S9: Validation of alternative splicing of ABCA7 exon 16 with Illumina RNAseq. IGV visualization of sequencing reads originating from lymphoblast RNA [3].Coordinates on chromosome 19 (hg19) are shown on top. The three panels below each correspond to a different individual and depict aligned sequencing reads (gray bars) as well as splicing events (blue connecting lines). At the bottom, a color code track corresponds to different nucleotides in the reference sequence and the canonical ABCA7 transcript (NM_019112) is shown in blue.

Using MinION cDNA sequencing we observed novel *ABCA7* isoforms which comprised alternatively spliced exon 16 (Figure S8). One particular splicing event encompassed the usage of a cryptic splice donor site 23bp upstream of the canonical splice donor site. In conjunction with a p.Glu709fs mutation (7bp deletion) this alternative splicing event can restore the transcript reading frame. Here, RNAseq analysis validates the existence of this isoform (red arrows). The upper RNAseq panel, corresponds to a p.Glu709fs carrier and depicts the limitations of RNAseq. No 7bp deletion is observed in exon 16, however, this is most likely a result of the low *ABCA7* coverage in RNAseq. Furthermore it cannot be determined wether the alterenative splicing observed in this panel is in phase with the mutation.

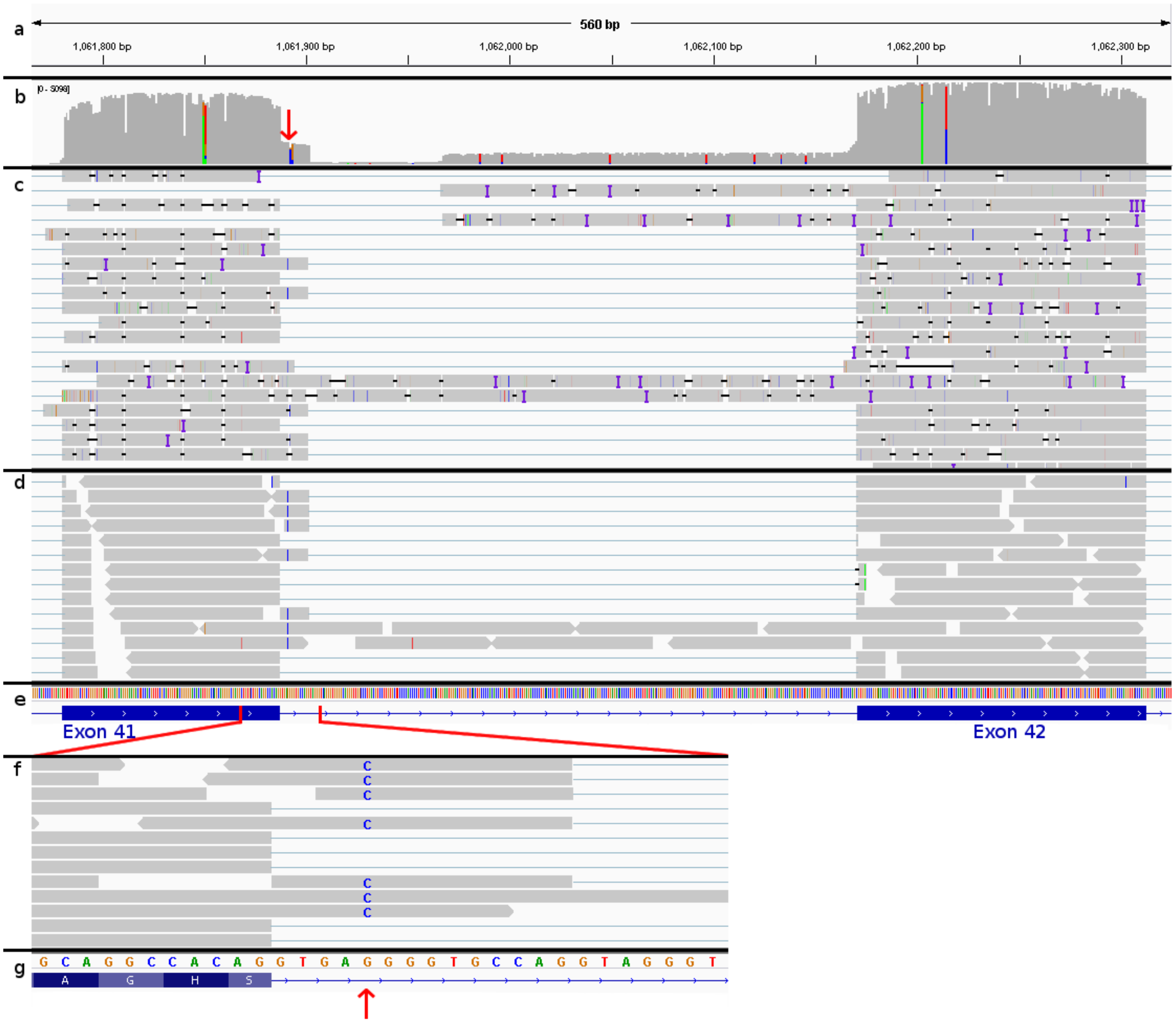

Figure S10: MinION cDNA sequencing and Illumina RNA sequencing of *ABCA7* c.5570+5G>C.

RNA and cDNA were obtained from c.5570+5G>C carrying patient lymphoblasts. Sequencing results are visualized with IGV. **(a)** Coordinates on chromosome 19 (hg19) and location of the PTC mutation (red arrow), corresponding to features in panels b, c, d, and e. **(b)** Coverage plot in which the height of each bar corresponds to the read depth at the corresponding position. Colors are shown depending on the observed nucleotides, and gray color indicates that the hg19 reference nucleotide was observed. **(c)** This panel represents MinION cDNA sequencing. Gray bars correspond to aligned sequences and blue lines denote splicing events. Several differentially spliced transcripts are observed: exon 41 skipping, 14bp intron 41 retention due to c.5570+5G>C, complete intron 41 retention and intron 41 retention starting from a cryptic splice acceptor site 204bp upstream of the canonical exon 42 splice acceptor. The accuracy of reads generated by the MinION platform, may be lower than Illumina sequencing (e.g. panel d), but is sufficient to align individual reads, which reveal a complex splicing pattern of this region. **(d)** (Partial) intron 41 retention is validated by RNAseq. This panel shows that to fully comprehend the splicing complexity, long reads (panel b) - instead of the short Illumina reads (gray segments) depicted

here - provide more clarity. Intron 41 retentions for instance are not spanned by the short reads, and it is therefore unknown whether these reads originate from a full or partial intron retention transcript. **(e)** An overview of the nucleotides (color code track) and *ABCA7* gene layout (blue). The red lines denote a region that was analyzed at higher depth in panels e and f. **(f)** Zoomed in RNAseq reads confirm the c.5570+5G>C[C]-allele as the cause of 14bp intron retention. **(g)** The reference nucleotides, amino acids to which the reads in panel e align, and location of c.5570+5G>C (red arrow).

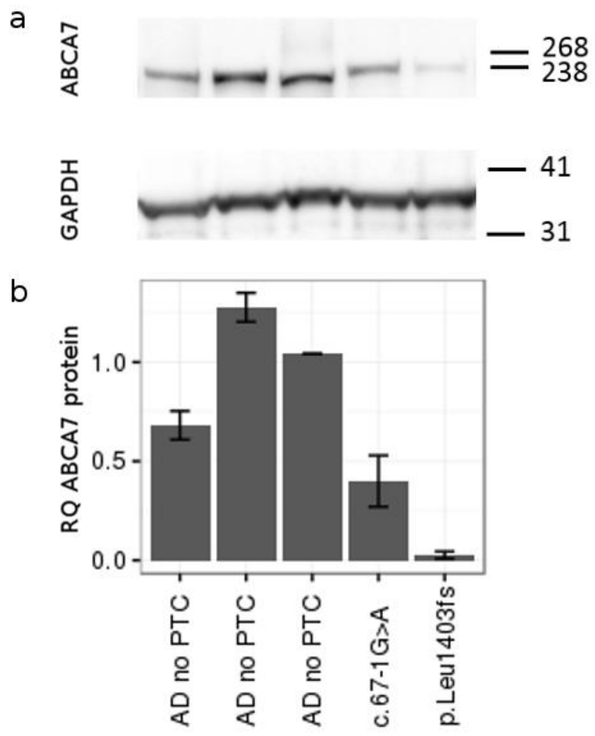

Figure S11: Hippocampal ABCA7 protein quantification in AD patients with or without an *ABCA7* PTC mutation. (a) Western blotting (Method S1) was performed on hippocampal brain tissue from three AD patients without an *ABCA7* PTC mutation (lanes 1, 2, and 3) and two carriers: c.67-1G>A (lane 4) and p.Leu1403fs (lane 5). A protein band is detected at approximately 234kDa, which corresponds to full length ABCA7 protein (ENSP00000414062). In addition, immunodetection of glyceraldehyde 3-phosphate dehydrogenase (GAPDH) is shown below. (b) Relative quantity (RQ) of ABCA7 is normalized by GAPDH signal strength. Western blotting was repeated twice.

Supplemental references

1. Allen M, Lincoln SJ, Corda M, Watzlawik JO, Carrasquillo MM, Reddy JS, et al. (2017) *ABCA7* loss-of-function variants, expression, and neurologic disease risk. Neurol Genet 3:e126. doi: 10.1212/NXG.0000000000000126

2. Cuyvers E, De Roeck A, Van den Bossche T, Van Cauwenberghe C, Bettens K, Vermeulen S, et al. (2015) Mutations in ABCA7 in a Belgian cohort of Alzheimer's disease patients: a targeted resequencing study. Lancet Neurol 14:814–822. doi: 10.1016/S1474-4422(15)00133-7

3. Verheijen J, Van den Bossche T, van der Zee J, Engelborghs S, Sanchez-Valle R, Lladó A, et al. (2016) A comprehensive study of the genetic impact of rare variants in SORL1 in European early-onset Alzheimer's disease. Acta Neuropathol 132:213–224. doi: 10.1007/s00401-016-1566-9
